# Combining multiomics and drug perturbation profiles to identify muscle-specific treatments for spinal muscular atrophy

**DOI:** 10.1172/jci.insight.149446

**Published:** 2021-07-08

**Authors:** Katharina E. Meijboom, Viola Volpato, Jimena Monzón-Sandoval, Joseph M. Hoolachan, Suzan M. Hammond, Frank Abendroth, Olivier G. de Jong, Gareth Hazell, Nina Ahlskog, Matthew J.A. Wood, Caleb Webber, Melissa Bowerman

**Affiliations:** 1 Department of Physiology, Anatomy and Genetics, University of Oxford, Oxford, United Kingdom.; 2 Gene Therapy Center, University of Massachusetts Medical School, Worcester, Massachusetts, USA.; 3 UK Dementia Research Institute, Cardiff University, Cardiff, United Kingdom.; 4 School of Medicine, Keele University, Staffordshire, United Kingdom.; 5 Department of Paediatrics, John Radcliffe Hospital and; 6 MDUK Oxford Neuromuscular Centre, University of Oxford, United Kingdom.; 7 Medical Research Council, Laboratory of Molecular Biology, Cambridge, United Kingdom.; 8 Institute of Chemistry, Philipps-University of Marburg, Marburg, Germany.; 9 Department of Pharmaceutics, Utrecht Institute for Pharmaceutical Sciences (UIPS), Faculty of Science, Utrecht University, Utrecht, Netherlands.; 10 Wolfson Centre for Inherited Neuromuscular Disease, RJAH Orthopaedic Hospital, Oswestry, United Kingdom.

**Keywords:** Muscle Biology, Neuroscience, Bioinformatics, Drug therapy, Genetic diseases

## Abstract

Spinal muscular atrophy (SMA) is a neuromuscular disorder caused by loss of survival motor neuron (SMN) protein. While SMN restoration therapies are beneficial, they are not a cure. We aimed to identify potentially novel treatments to alleviate muscle pathology combining transcriptomics, proteomics, and perturbational data sets. This revealed potential drug candidates for repurposing in SMA. One of the candidates, harmine, was further investigated in cell and animal models, improving multiple disease phenotypes, including lifespan, weight, and key molecular networks in skeletal muscle. Our work highlights the potential of multiple and parallel data-driven approaches for the development of potentially novel treatments for use in combination with SMN restoration therapies.

## Introduction

Spinal muscular atrophy (SMA) is an autosomal recessive neuromuscular disorder (1) and the leading genetic cause of infant mortality (2). The major pathological components of the disease are the selective loss of spinal cord α motor neurons, progressive muscle denervation (3), and skeletal muscle atrophy (4). SMA is caused by mutations in the survival motor neuron 1 (*SMN1*) gene (5). SMN protein is ubiquitously expressed, and complete loss is lethal (6). However, humans have a near-identical centromeric copy of the *SMN1* gene, termed *SMN2*, in which a single nucleotide change (C to T) in exon 7 (7) results in the exclusion of exon 7 from approximately 90% of the mature transcript (8). The resulting protein is unstable and gets rapidly degraded (9). Patients can have a varying number of *SMN2* copies, which correlates with disease severity, as each *SMN2* copy retains the ability to produce approximately 10% of functional full-length (FL) protein (10, 11).

The first SMN restoration treatments, Spinraza, Zolgensma, and Evrysdi, have recently been approved by the US Food and Drug Administration (FDA) and the European Medicines Agency (EMA). Spinraza (12) is an antisense oligonucleotide (ASO) that promotes *SMN2* exon 7 inclusion (13) and is administered by lumbar puncture, Zolgensma delivers *SMN1* cDNA via an adeno-associated virus 9 (14) and is administered i.v., and Evrysdi is a small molecule that increases *SMN2* exon 7 inclusion and is administered orally (15). While these treatments have changed the SMA therapeutic landscape, they unfortunately fall short of representing a cure (16–18). There is, therefore, a present need for SMN-independent therapies that could be used in combination with SMN restoration treatments to provide a longer-lasting and more effective therapeutic management of SMA pathology in patients (16–18).

Skeletal muscle pathology is a clear contributor to SMA disease manifestation and progression, and improving muscle health could have significant benefits for patients (19). Here, we used an in-depth and parallel approach combining proteomics, transcriptomics, and the drug perturbational data set Connectivity Map (CMap; refs. 20, 21) to identify differentially expressed (DE) transcripts and proteins in skeletal muscle of the severe Taiwanese *Smn^–/–^;SMN2* SMA mice (22) that could potentially be restored by known and available pharmacological compounds. This strategy uncovered several potential therapeutic candidates, including harmine, which was further evaluated in cell and animal models, showing an ability to restore molecular networks and improve several disease phenotypes, including lifespan and weight. Our study highlights the tremendous potential of intersecting disease multiomics with drug perturbational responses to identify therapeutic compounds capable of modulating dysfunctional cellular and molecular networks to ameliorate SMA phenotypes.

## Results

### Early restoration of SMN in SMA mice restores muscle protein and transcript expression.

**Figure 1:**
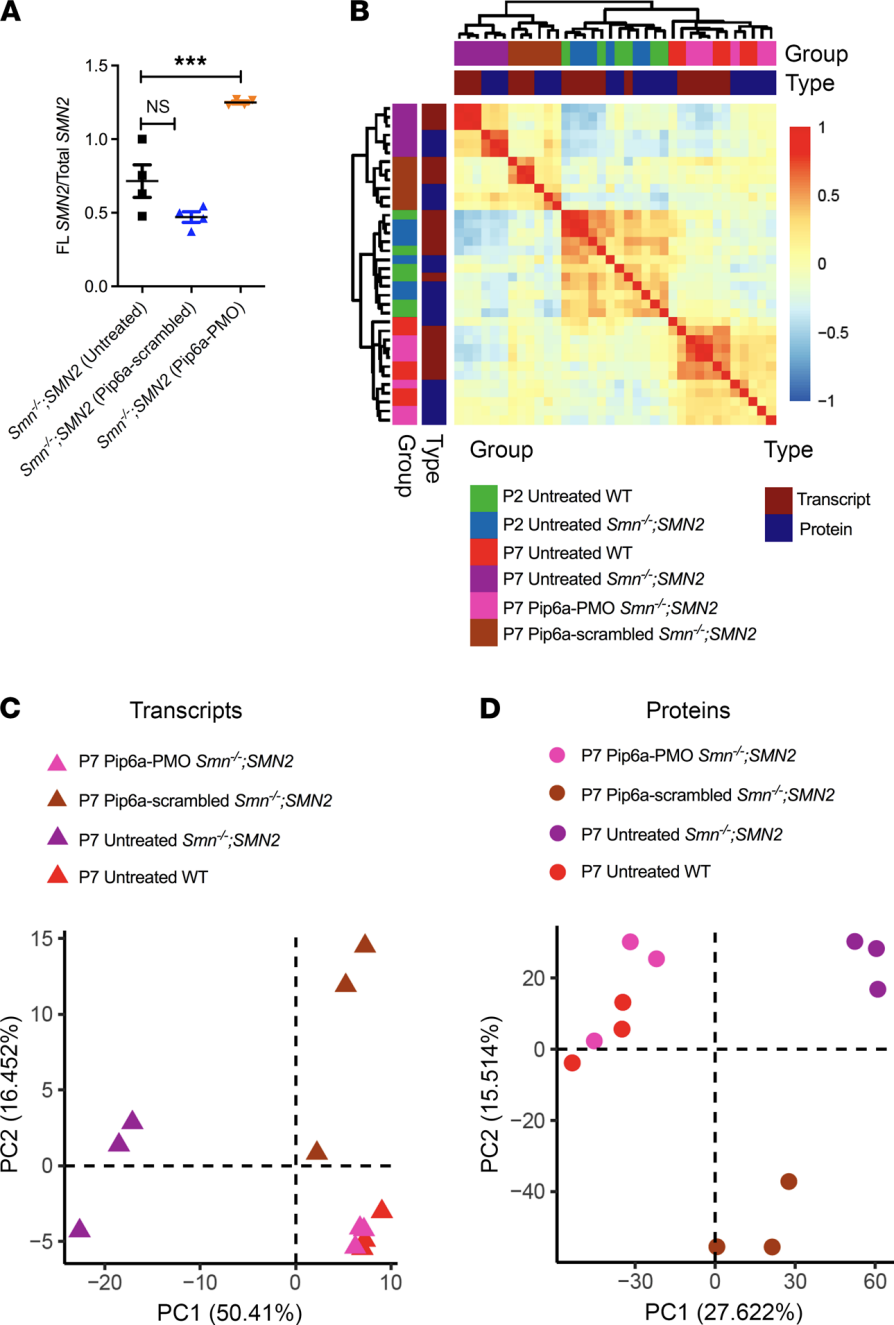
Restoration of protein and transcript expression in skeletal muscle of SMA mice following early SMN restoration treatment. *Smn^–/–^;SMN2* mice received a facial i.v. injection at P0 and P2 of Pip6a-scrambled or Pip6a-PMO (10 μg/g). The *tibialis anterior* was harvested from P2 untreated *Smn^–/–^;SMN2* and WT mice; from P7 untreated, Pip6a-scrambled-treated, and Pip6a-PMO-treated *Smn^–/–^;SMN2* mice; and from P7 untreated WT mice. (**A**) Comparison of the ratio of full-length (FL) *SMN2* over total *SMN2* quantified by qPCR between P7 untreated, Pip6a-scrambled-treated, and Pip6a-PMO–treated *Smn^–/–^;SMN2* mice. Data are shown as a scatter plot and are represented as mean ± SEM; *n =* 4 animals per experimental group, 1-way ANOVA followed by a Dunnett’s multiple comparisons test, F ratio (F) = 34.88, degrees of freedom (df) = 11, ***P < 0.001. (**B**) Heatmap of the transcriptomic and proteomic expression profiles measured by the Pearson correlation between each pair of samples (after the removal of the first principal component). (**C**) First 2 principal components based on transcriptomic profiles of P7 untreated WT mice, untreated *Smn^–/–^;SMN2* mice, Pip6a-PMO–treated *Smn^–/–^;SMN2* mice, and Pip6a-scrambled *Smn^–/–^;SMN2* mice. (**D**) First 2 principal components based on proteomic profiles of P7 untreated WT mice, untreated *Smn^–/–^;SMN2* mice, Pip6a-PMO–treated *Smn^–/–^;SMN2* mice, and Pip6a-scrambled *Smn^–/–^;SMN2* mice.

We first set out to determine the effect of early SMN restoration on the proteomic and transcriptomic profiles of SMA skeletal muscle, with the intent to design therapeutic strategies against the genes and proteins that remained unchanged. To do so, the severe Taiwanese *Smn^–/–^;SMN2* SMA mouse model (22) received a facial i.v. injection at P0 and P2 of the previously described Pip6a-phosphordiamidate morpholino oligomer (Pip6a-PMO) or Pip6a-scrambled pharmacological compounds (10 μg/g; ref. 23, 24). Pip6a is a cell-penetrating peptide (CPP) conjugated either to an *SMN2* exon 7 inclusion-promoting ASO (PMO) or a scrambled ASO (23, 24). We have previously reported that administration of Pip6a-PMO to newborn *Smn^–/–^;SMN2* mice led to increased SMN protein levels in numerous tissues, including skeletal muscle, and a concomitant 40-fold increase in survival (23). We harvested the *tibialis anterior* (TA) from P2 (presymptomatic) untreated *Smn^–/–^;SMN2* and WT mice, P7 (symptomatic) untreated *Smn^–/–^;SMN2* and WT mice, and P7 Pip6a-scrambled *Smn^–/–^;SMN2* and Pip6a-PMO–treated *Smn^–/–^;SMN2* mice. TAs were then cut in 2, whereby one half was used for transcriptomics (whole-transcript array assay) and the other for proteomics (liquid chromatography–mass spectrometry; LC-MS). Quantitative PCR (qPCR) analysis of the ratio of FL *SMN2* over total *SMN2* confirms a significant increase in FL *SMN2* expression in P7 Pip6a-PMO–treated *Smn^–/–^;SMN2* mice compared with age-matched untreated and Pip6a-scrambled–treated *Smn^–/–^;SMN2* mice (Figure 1A).

Despite differences between the transcriptomic and proteomic methodologies, highlighted by hierarchical clustering and combined Principal Component Analysis (PCA; Supplemental Figure 1; supplemental material available online with this article; https://doi.org/10.1172/jci.insight.149446DS1), we were able to find clear separation of experimental groups and agreement between transcriptomic and proteomic profiles once the variance attributed to the differences in methodologies was removed (Figure 1B). At P7, we observed a clear separation of *Smn^–/–^;SMN2* and WT samples, where only Pip6a-PMO–treated *Smn^–/–^;SMN2* mice clustered with WT (Figure 1B and Supplemental Figure 2). We also found that P2 *Smn^–/–^;SMN2* and WT samples clustered together (Figure 1B and Supplemental Figure 2), suggesting that overt disease cannot be detected in omics readouts at this early stage. In the PCA of P7 samples only (Figure 1C for transcriptomics and Figure 1D for proteomics), we noted clustering of P7 Pip6a-PMO–treated *Smn^–/–^;SMN2* mice with untreated P7 WT animals, suggesting a significant restoration of both transcriptomic and proteomic expression profiles. Surprisingly, we also detected segregation of Pip6a-scrambled–treated samples at both transcriptomics and proteomics levels, revealing that presence of the CPP itself impacted transcription and translation (Figure 1, C and D, and Supplemental Table 1). Importantly, both the combined and separate analyses of transcriptomic and proteomic data allowed us to identify a robust SMA disease signature in muscle and a Pip6a-PMO treatment efficacy signature. Indeed, identification of DE genes and proteins revealed that early induction of FL *SMN* expression by Pip6a-PMO normalized the expression of all transcripts and all but 11 proteins in the TA of *Smn^–/–^;SMN2* mice (Tables 1 and 2). Of note, one of the proteins that remained significantly downregulated is SMN itself (Table 2), which is in contrast with the complete normalization of FL *SMN2* transcript levels (Figure 1A) and perhaps due to distinct regulation of SMN RNA and protein stability (25, 26) that might be impacted differently during development — in this case, prior to Pip6a-PMO–mediated SMN restoration. Nevertheless, this increase is sufficient to rescue the disease phenotype, which is aligned with previous reports of an SMN threshold, whereby a normal phenotype has been observed in mice with as little as 30% SMN protein when compared with WT levels (27).

Our in-depth molecular profiling thus demonstrates for the first time, to the best of our knowledge, that increasing FL *SMN2* in neonatal SMA mice almost completely normalizes muscle transcripts and proteins, highlighting at the molecular level the potential treatment benefits arising from early intervention.

### CMap perturbational profiles identify potential novel non-SMN treatments.

**Figure 2:**
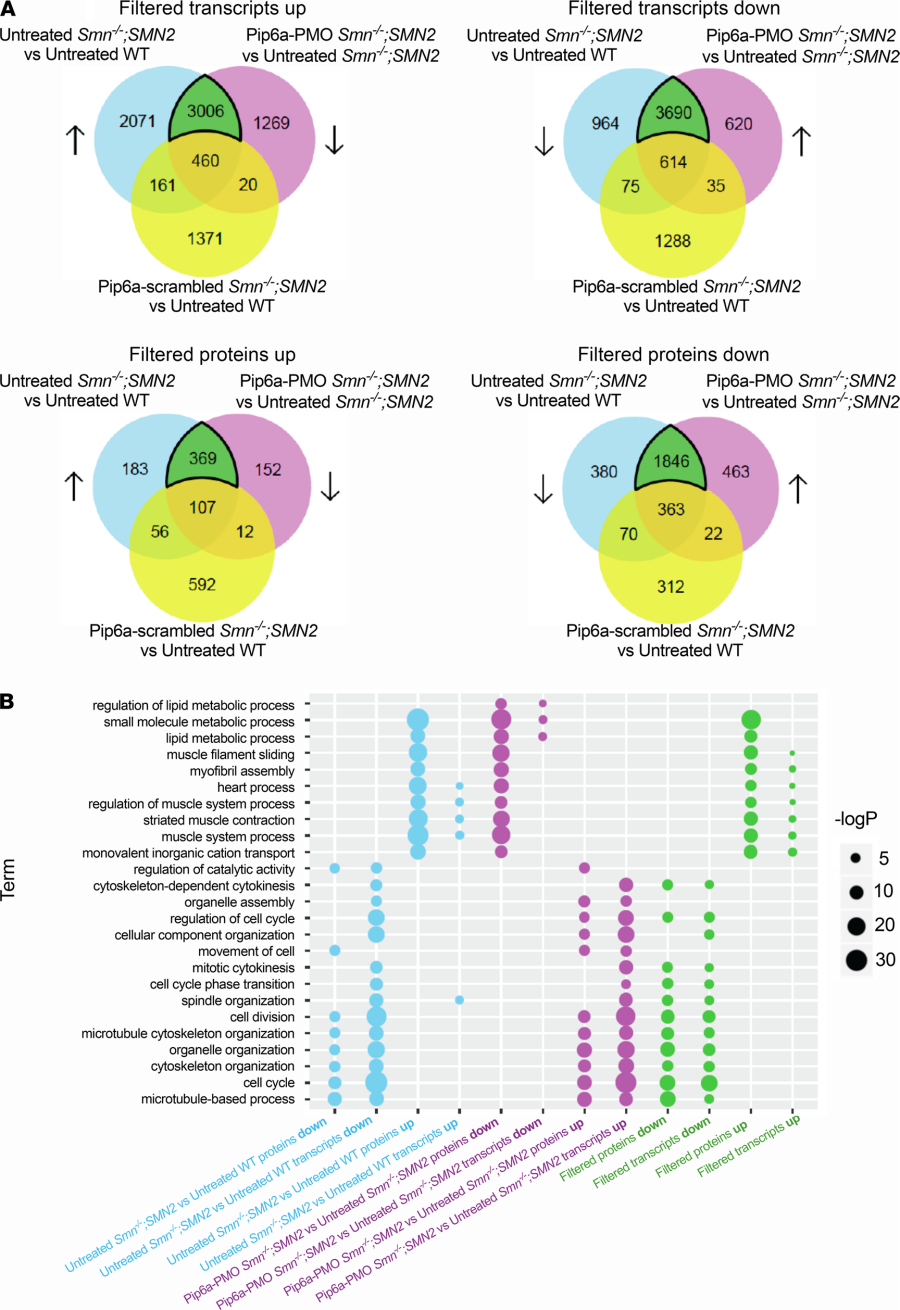
Identification of disease signal reversed by treatment with Pip6a-PMO by removing the effect of Pip6a scrambled at transcriptomic and proteomic levels. (**A**) Venn diagrams show the number of transcripts (top) and proteins (bottom) differentially expressed (DE) between untreated *Smn^–/–^;SMN2* and untreated WT mice, reversed by treatment with Pip6a-PMO and not DE between Pip6a-scrambled–treated *Smn^–/–^;SMN2* mice and untreated WT animals. Filtered signatures were named according to the increase (up) or decrease (down) expression in untreated *Smn^–/–^;SMN2* mice compared with untreated WT animals and are highlighted in the green area of the Venn diagrams. (**B**) Set of enriched gene ontology (GO) biological processes that show similarity across comparisons. GO enrichment analysis was performed separately for transcripts and proteins that were DE between untreated *Smn^–/–^;SMN2* and untreated WT mice (blue), DE between Pip6a-PMO-treated *Smn^–/–^;SMN2* and untreated *Smn^–/–^;SMN2* mice (purple), and part of the filtered signatures described in **A** (green).

We used the transcriptomic and proteomic profiles of the *Smn^–/–^;SMN2* mice treated with Pip6a-PMO to find drugs that induced similar transcriptional patterns using the CMap resource (20, 28). Briefly, CMap is a database where gene expression profiles of human cell lines treated with different drugs are collected, therefore providing a resource for drug repurposing studies. Specifically, by selecting drugs that induce gene expression profiles that are inverse (or anticorrelated) to disease-associated gene expression profiles, it is possible to identify new candidate therapeutics with the potential to counteract the disease effects under investigation. Thus, we firstly generated a filtered and reversed disease signature for both transcriptomics and proteomics data by excluding the transcripts and proteins restored by Pip6a-scrambled (Pip6a-scrambled–treated *Smn^–/–^;SMN2* versus untreated WT) from the overlap between disease (untreated *Smn^–/–^;SMN2* versus untreated WT) and Pip6a-PMO (Pip6a-PMO treated *Smn^–/–^;SMN2* versus untreated *Smn^–/–^;SMN2*) (Figure 2A). These filtered sets of transcripts and proteins show a significant overlap between different data types (Supplemental Figure 3) and a greater similarity at the level of enriched pathways when compared with nonfiltered sets (Figure 2B). A complete list of enriched gene ontology (GO) biological processes across all tested comparisons (transcripts and proteins) is compiled in Supplemental Table 2.

The top 10 pharmacological compounds from CMap showed a reversed pattern of expression for the disease signature and a similar expression pattern to that observed with Pip6a-PMO treatment, listed in Table 3. Importantly, a subset of these drugs — namely, salbutamol (29) and alsterpaullone (30) — have already been considered for SMA treatment, highlighting the capability of this analytic approach to identify relevant therapeutic options for SMA.

Our bioinformatic analysis, therefore, revealed that the Pip6a peptide itself led to several molecular changes in skeletal muscle, underscoring the importance of including such controls to avoid erroneous interpretations. Here, the generation of filtered data sets that excluded proteins and transcripts modulated by the Pip6a peptide only, allowed us to confidently identify transcripts, proteins, and biological pathways selectively restored by increased SMN levels and relevant candidate drugs predicted to have similar activities. Thus, our strategy of combining transcriptomics, proteomics, and drug perturbational data sets has resulted in the generation of a list of several pharmacological compounds with the potential to restore muscle health in SMA.

### Harmine displays predicted activity on candidate reporter genes in a cell- and dose-dependent manner.

To further validate our combined bioinformatics and drug repurposing approach, we chose to evaluate the potential of harmine (chemically akin to harmol), which is a drug that has been identified by its CMap profile but not previously evaluated for SMA and which is present in several proteomic and transcriptomic signatures (Table 3). Harmine is an alkaloid isolated from the seeds of Peganum harmala, traditionally used for ritual and medicinal preparations (31, 32). Harmine has also demonstrated therapeutic benefits (33) in animal models of the motor neuron disease amyotrophic lateral sclerosis (ALS) (34) and the muscle disorder myotonic dystrophy type 1 (DM1; ref. 35).

**Figure 3:**
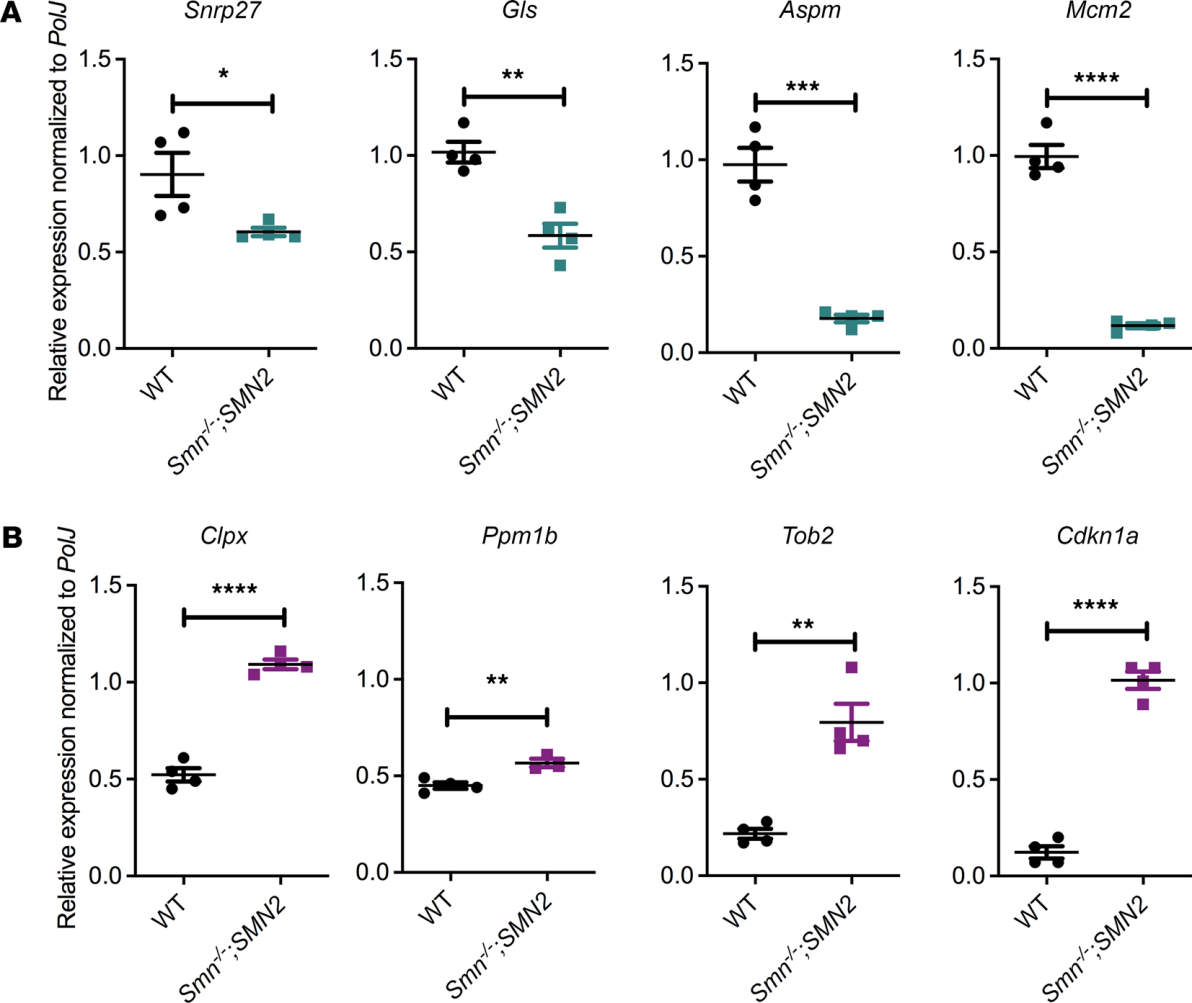
Harmine target genes, as predicted by CMap analyses, are aberrantly expressed in SMA muscle. (**A**) qPCR analysis of genes predicted to be significantly downregulated (*Snrnp27*, *Gl*s, *Aspm*, and *Mcm2*) in the TA of untreated P7 SMA *Smn^–/–^;SMN2* and WT mice. Data are shown as a scatter plot and are represented as mean ± SEM; *n =* 4 animals per experimental group, unpaired *t* test, df = 6 for all, *P =* 0.041 (*Snrnp27*), *P =* 0.0019 (*Gl*s), *P =* 0.0001 (*Aspm*), *P <* 0.0001 (*Mcm2)*. (**B**) qPCR analysis of genes predicted to be upregulated (*Clpx*, *Ppm1b*, *Tob2*, and *Cdkn1a*) in the TA of untreated P7 SMA *Smn^–/–^;SMN2* and WT mice. Data are shown as a scatter plot and are represented as mean ± SEM; *n =* 4 animals per experimental group, unpaired *t* test, df = 6 for all except *Ppm1b*, where df = 5; *P <* 0.0001 (*Clpx*), *P =* 0.0076 (*Ppm1b*), *P =* 0.0012 (*Tob2*), *P <* 0.0001 (*Cdkn1a*).

**Figure 4:**
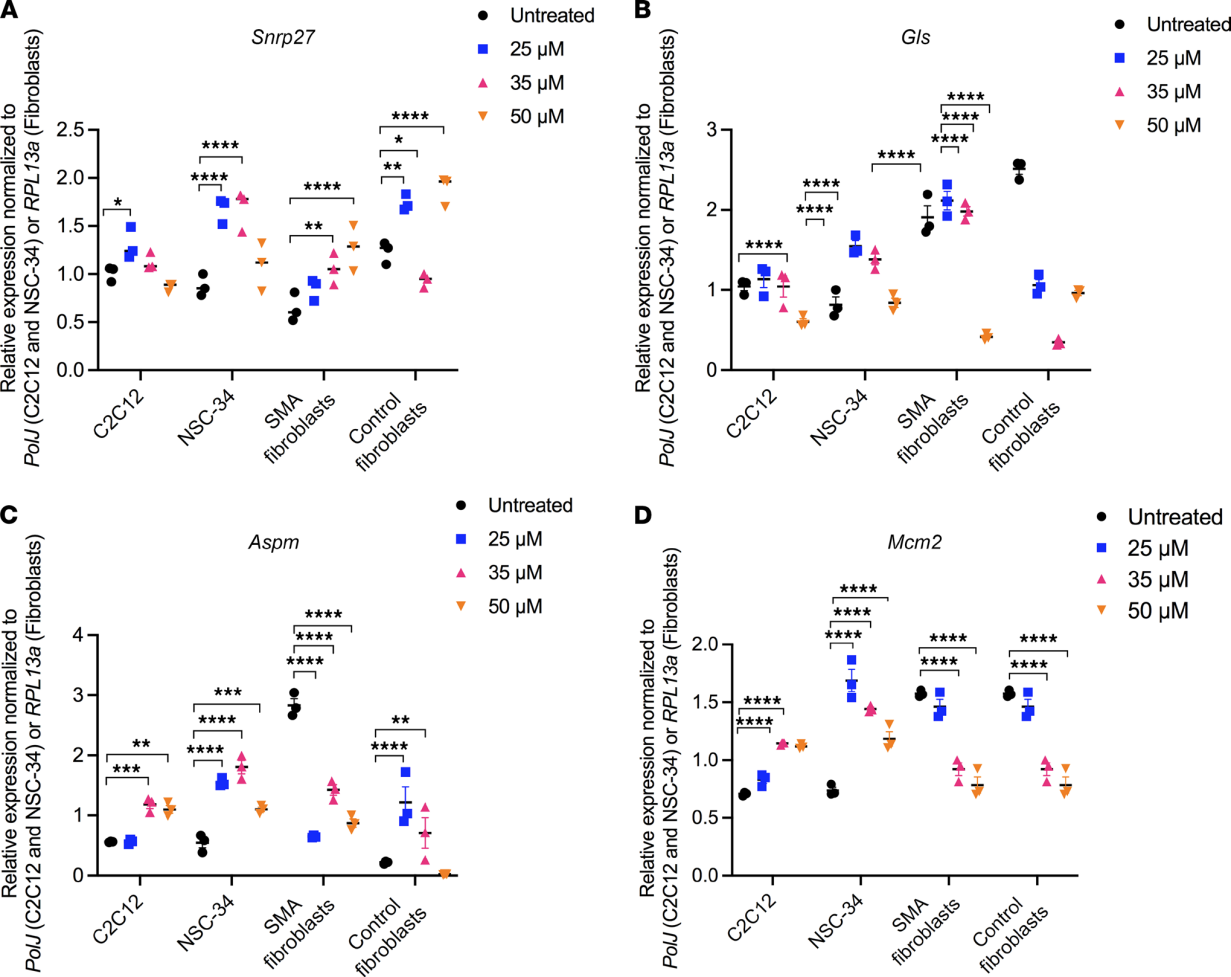
Harmine, as predicted by CMap analyses, is able to reverse the expression of genes significantly downregulated in SMA muscle in several cellular models. (**A**–**D**) C2C12, NSC-34, SMA patient fibroblasts, and control fibroblasts were treated with 25, 35, or 50 μM of harmine for 48 hours. Expression of *Snrnp27* (**A**), *Gl*s (**B**), *Aspm* (**C**), and *Mcm2* (**D**) was assessed by qPCR and compared with untreated cells. Data are shown as a scatter plot and are represented as mean ± SEM; *n =* 3 independent wells, 2-way ANOVA followed by uncorrected Fisher’s least significant difference (LSD), F = 20.20 (*Snrnp27*), F = 90.95 (*Gl*s), F = 14.16 (*Aspm*), F = 42.61 (*Mcm2*), df = 32 for all, *P < 0.05, **P < 0.01, ***P < 0.001, ****P < 0.0001.

**Figure 5:**
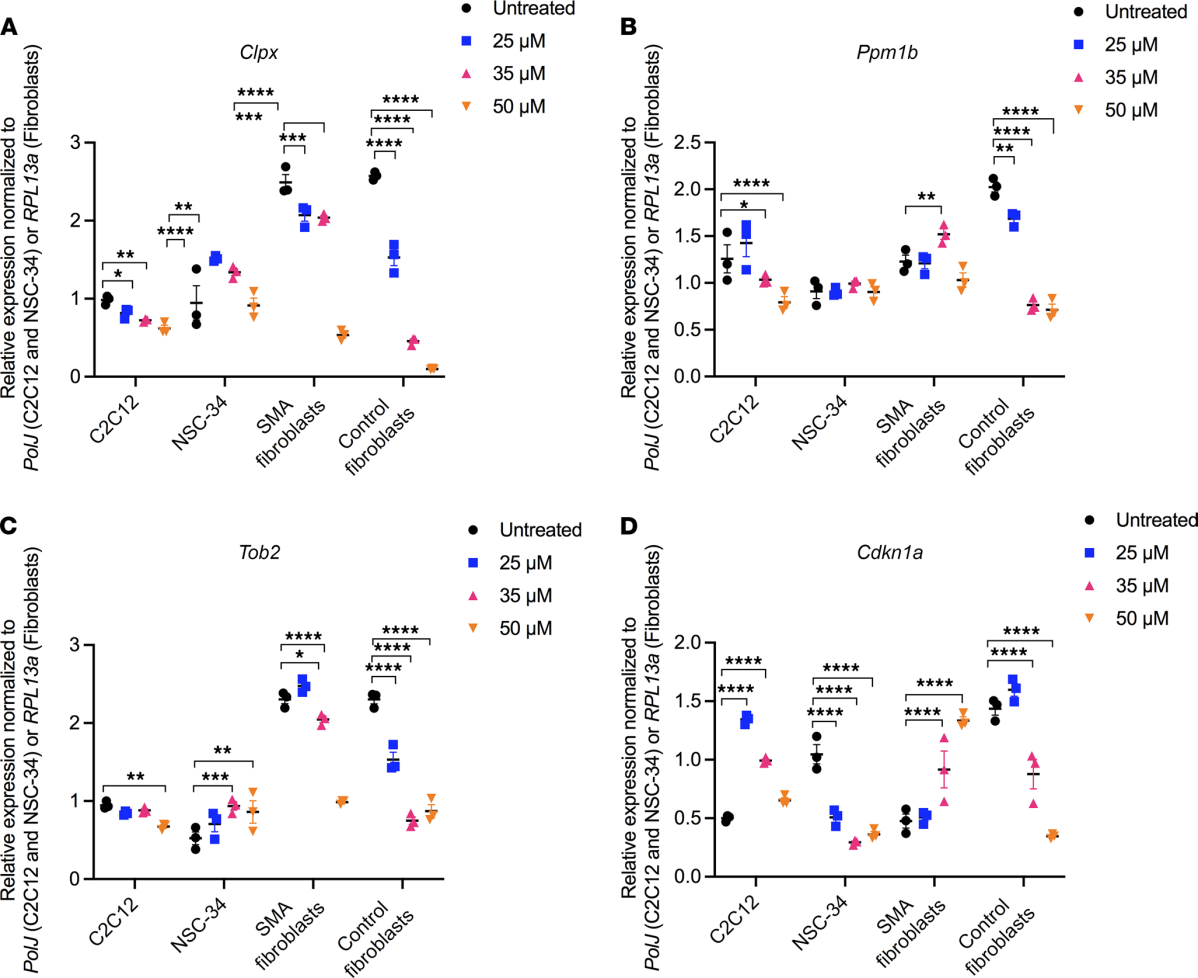
Harmine, as predicted by CMap analyses, is able to reverse the expression of genes significantly upregulated in SMA muscle in several cellular models. (**A**–**D**) C2C12s, NSC-34s, SMA patient fibroblasts, and control fibroblasts were treated with 25, 35, or 50 μM of harmine for 48 hours. Expression of *Clpx* (**A**), *Ppm1b* (**B**), *Tob2* (**C**), and *Cdkn1a* (**D**) was assessed by qPCR and compared with untreated cells. Data are shown as a scatter plot and are represented as mean ± SEM; *n =* 3 independent wells, 2-way ANOVA followed by uncorrected Fisher’s LSD, F = 182 (*Clpx*), F = 38.49 (*Ppm1b*), F = 78.17 (*Tob2*), F = 18.36 (*Cdkn1a*), df = 32 for all, *P < 0.05, **P < 0.01, ***P < 0.001, ****P < 0.0001.

We firstly evaluated the mRNA expression of the transcripts and proteins predicted to be dysregulated by the transcriptomics and proteomics data and to be normalized by harmine through the CMap analysis. We indeed confirmed by qPCR analysis that the genes snRNP U4/U6.U5 subunit 27 (*Snrnp27*), glutaminase (*Gls*), assembly factor for spindle microtubules (*Aspm*), and minichromosome maintenance complex component 2 (*Mcm2*) are significantly downregulated (Figure 3A), while caseinolytic mitochondrial matrix peptidase chaperone subunit X (*Clpx*), protein phosphatase, Mg^2+^/Mn^2+^-dependent 1B (*Ppm1b*), transducer of ERBB2, 2 (*Tob2*), and cyclin-dependent kinase inhibitor 1A (*Cdkn1a*) are significantly upregulated (Figure 3B) in the TA of P7 *Smn^–/–^;SMN2* mice compared with WT animals. We then evaluated the ability of harmine to impact the expression of these genes by treating C2C12 myoblasts, NSC-34 neuronal-like cells, SMA patient fibroblasts, and control fibroblasts with 25, 35, and 50 μM of the drug for 48 hours. Our bioinformatic analysis predicted that harmine would increase the expression of *Snrnp27*, *Gls*, *Aspm*, and *Mcm2*, and we observed an increased expression of these genes, albeit in a cell- and dose-dependent manner (Figure 4, A–D). Indeed, some cell types displayed a decreased expression of the candidate reporter genes (e.g., *Aspm* in SMA patient fibroblasts; Figure 4C), and some cell types demonstrated an increased expression only at a specific concentration of the drug (e.g., *Gls* in NSC-34s; Figure 4B). Similar results were obtained when evaluating the expression of *Clpx*, *Ppm1b*, *Tob2*, and *Cdkn1a* — genes predicted to be downregulated by harmine (Figure 5, A–D). For the most part, harmine decreased the expression of these genes, with some exceptions where expression was in fact increased (e.g., *Cdkn1a* in C2C12s; Figure 5D) or decreased only at certain doses (e.g., *Tob2* in SMA patient fibroblasts; Figure 5C). Our observed cell- and dose-dependent pharmacological activity of harmine most likely reflects that the CMap resource is based on data from human cancer cell lines (20, 28). In addition, harmine displayed inhibitory effects on proliferation and viability at the higher doses in C2C12s and NSC-34s (Supplemental Figure 4), which perhaps influenced the differential effects of low and high concentrations in some cell types.

We were thus able to demonstrate the strength of our combined bioinformatics and drug repurposing approach by selecting harmine for additional proof-of-concept investigations. Indeed, we confirmed the predicted dysregulation of several candidate reporter genes in skeletal muscle of symptomatic SMA mice and observed a cell- and concentration-dependent modulation of their expression by harmine.

### Administration of harmine to SMA mice ameliorates disease phenotypes.

**Figure 6:**
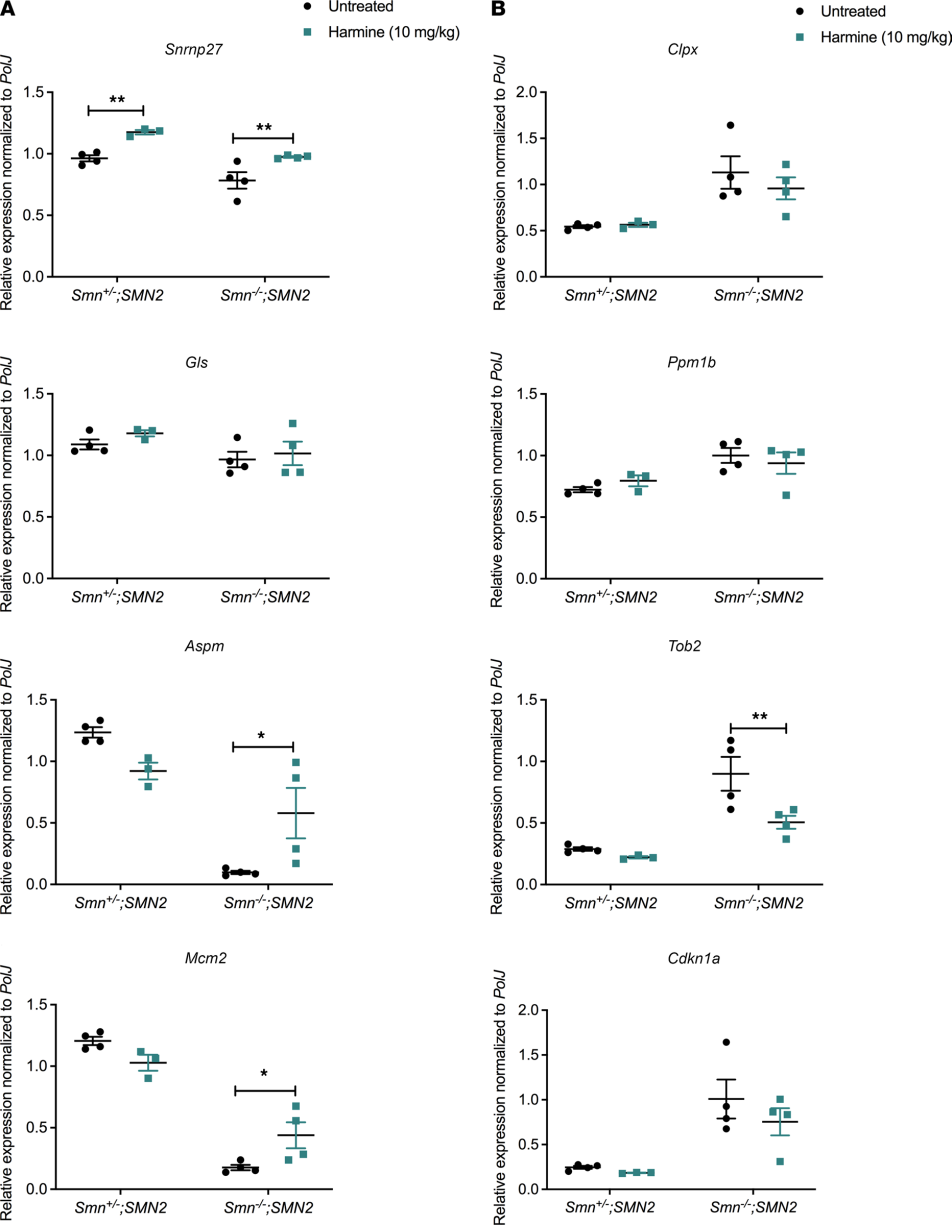
Administration of harmine to SMA mice partially restores the expression of target genes, as predicted by CMap analyses. All treated animals received a daily dose of harmine (10 mg/kg, diluted in 0.9% saline) by gavage starting at P0. (**A**) qPCR analysis of *Snrnp27*, *Gl*s, *Aspm,* and *Mcm2* in triceps of P7 untreated and harmine-treated *Smn^–/–^;SMN2* SMA mice and *Smn^+/–^;SMN2* control littermates. Data are shown as a scatter plot and are represented as mean ± SEM, *n =* 4 animals per experimental group except for harmine-treated *Smn^+/–^;SMN2* where *n =* 3, 2-way ANOVA followed by a Sidak’s multiple comparisons test, F = 25.77 (*Snrnp27*), F = 1.103 (*Gl*s), F = 0.5143 (*Aspm*), F = 0.3992 (*Mcm2*), df = 11 for all, *P < 0.05, **P < 0.01. (**B**) qPCR analysis of *Clpx*, *Ppm1b*, *Tob2*, and *Cdkn1a* in triceps of P7 untreated and harmine-treated *Smn^–/–^;SMN2* SMA mice and *Smn^+/–^;SMN2* control littermates. Data are shown as a scatter plot and are represented as mean ± SD; *n =* 4 animals per experimental group except for harmine-treated *Smn^+/–^;SMN2* where *n =* 3, 2-way ANOVA followed by a Sidak’s multiple comparisons test, F = 0.4275 (*Clpx*), F = 0.006960 (*Ppm1b*), F = 8.167 (*Tob2*), F = 1.195 (*Cdkn1a*), df = 11 for all, **P < 0.01.

To further evaluate the potential therapeutic effects of harmine in vivo, we administered it daily to *Smn^–/–^;SMN2* mice and *Smn^+/–^;SMN2* control littermates by gavage (10 mg/kg diluted in 0.9% saline) starting at P0. The 10 mg/kg dose was chosen based on its previous demonstrations of activity and safety in rodents (36). We first evaluated the effects of harmine on the expression of the candidate reporter genes predicted to be restored by harmine. Of the genes predicted to be upregulated by harmine (*Snrnp27*, *Gls*, *Aspm*, and *Mcm2*)*,* daily harmine administration increased the expression of *Snrnp27* in both *Smn^–/–^;SMN2* SMA mice and *Smn^+/–^;SMN2* control littermates and of *Aspm* and *Mcm2* in SMA muscle only, compared with untreated cohorts (Figure 6A). Of the genes predicted to be downregulated by harmine (*Clpx*, *Ppm1b, Cdkn1a*, and *Tob2*), harmine only reduced the expression of *Tob2* in SMA mice compared with untreated animals (Figure 6B). Of note, while the *Smn^+/–^;SMN2* mice are healthy littermates in terms of lifespan and reproductive abilities, they nevertheless have reduced levels of Smn, which in itself has been demonstrated to impact certain phenotypic features (e.g., tail and ear necrosis, metabolism, gene expression; refs. 22, 37). As such, comparisons were performed between untreated and harmine-treated animals of the same genotype, allowing us to determine if the effects were SMA dependent or SMA independent, without the addition of a potential compounding factor.

**Figure 7:**
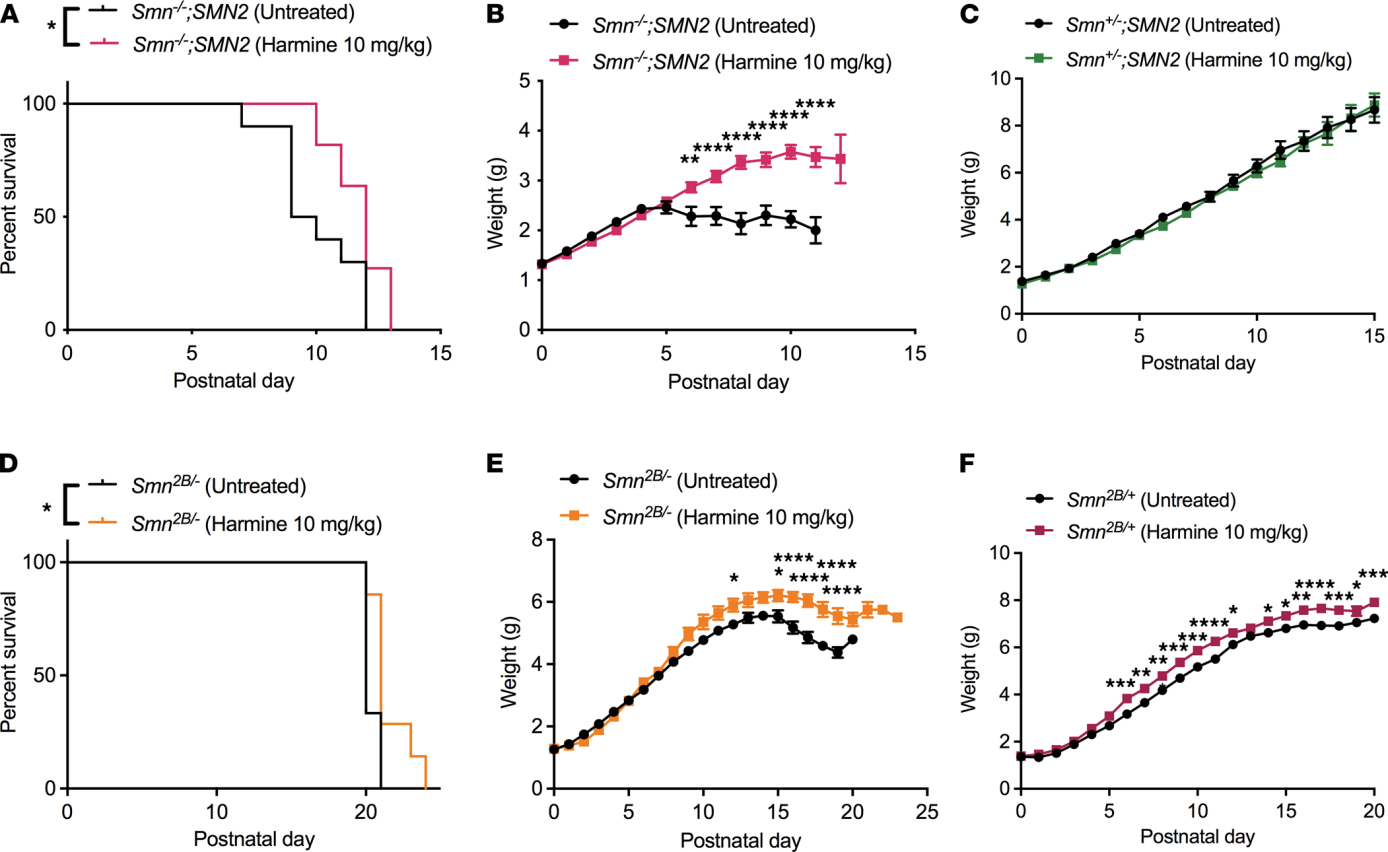
Administration of harmine to SMA mice improves weight and survival. All treated animals received a daily dose of harmine (10 mg/kg, diluted in 0.9% saline) by gavage starting at P0. (**A**) Survival curves of untreated and harmine-treated *Smn^–/–^;SMN2* mice. Kaplan-Meier survival curve is shown, with *n =* 10 for untreated *Smn^–/–^;SMN2* mice, *n =* 11 for harmine-treated *Smn^–/–^;SMN2* mice, Log-rank (Mantel-Cox) test, *P = 0.0211. (**B**) Daily weights of untreated and harmine-treated *Smn^–/–^;SMN2* mice. Data are represented as mean ± SEM; *n =* 10 for untreated *Smn^–/–^;SMN2* mice, *n =* 11 for harmine-treated *Smn^–/–^;SMN2* mice, 2-way ANOVA followed by a Sidak’s multiple comparisons test, F = 95.70, df = 202, **P < 0.01, ****P < 0.0001. (**C**) Daily weights of untreated and harmine-treated *Smn^+/–^;SMN2* mice. Data are represented as mean ± SEM; *n =* 13 for untreated *Smn^+/–^;SMN2* mice, *n =* 15 for harmine-treated *Smn^+/–^;SMN2* mice, 2-way ANOVA followed by a Sidak’s multiple comparisons test, F = 2.897, df = 398. (**D**) Survival curves of untreated and harmine-treated *Smn^2B/–^* mice. Kaplan-Meier survival curves are shown, with *n =* 9 for untreated *Smn^2B/–^* mice, *n =* 7 for harmine-treated *Smn^2B/–^* mice, log-rank (Mantel-Cox) test, *P = 0.0221. (**E**) Daily weights of untreated and harmine-treated *Smn^2B/–^* mice. Data are represented as mean ± SEM, *n =* 9 for untreated *Smn^2B/–^* mice, *n =* 7 for harmine-treated *Smn^2B/–^* mice, 2-way ANOVA followed by a Sidak’s multiple comparisons test, F = 96.25, df = 287, *P < 0.05, ****P < 0.0001. (**F**) Daily weights of untreated and harmine-treated *Smn^2B/+^* mice. Data are represented as mean ± SEM, *n =* 13 for untreated *Smn^2B/+^* mice, *n =* 8 for harmine-treated *Smn^2B/+^* mice, 2-way ANOVA followed by a Sidak’s multiple comparisons test, F = 206.3, df = 399, *P < 0.05, **P < 0.01, ***P < 0.001, ****P < 0.0001.

We next assessed the effect of harmine on disease progression and found a significant increase in survival of harmine-treated *Smn^–/–^;SMN2* mice compared with untreated *Smn^–/–^;SMN2* animals (Figure 7A). Harmine administration also improved weights of treated *Smn^–/–^;SMN2* mice compared with untreated *Smn^–/–^;SMN2* animals (Figure 7B), while it did not impact the weights of *Smn^+/–^;SMN2* control littermates (Figure 7C). An intermediate SMA mouse model, termed *Smn^2B/–^* (27), was also treated with harmine. Harmine administration to *Smn^2B/–^* mice similarly resulted in a significant increase in survival compared with untreated *Smn^2B/–^* animals (Figure 7D), albeit to a lesser extent, most likely due to the fact that the treated animals developed tremors and needed to be culled. Tremors have indeed been reported in animal studies of long-term harmine administration (38–40). Nevertheless, harmine significantly increased the weights of treated *Smn^2B/–^* mice compared with untreated *Smn^2B/–^* animals (Figure 7E). Interestingly, harmine also had a small but significant impact on the weights of treated *Smn^2B/+^* control littermates compared with untreated *Smn^2B/+^* animals (Figure 7F).

**Figure 8:**
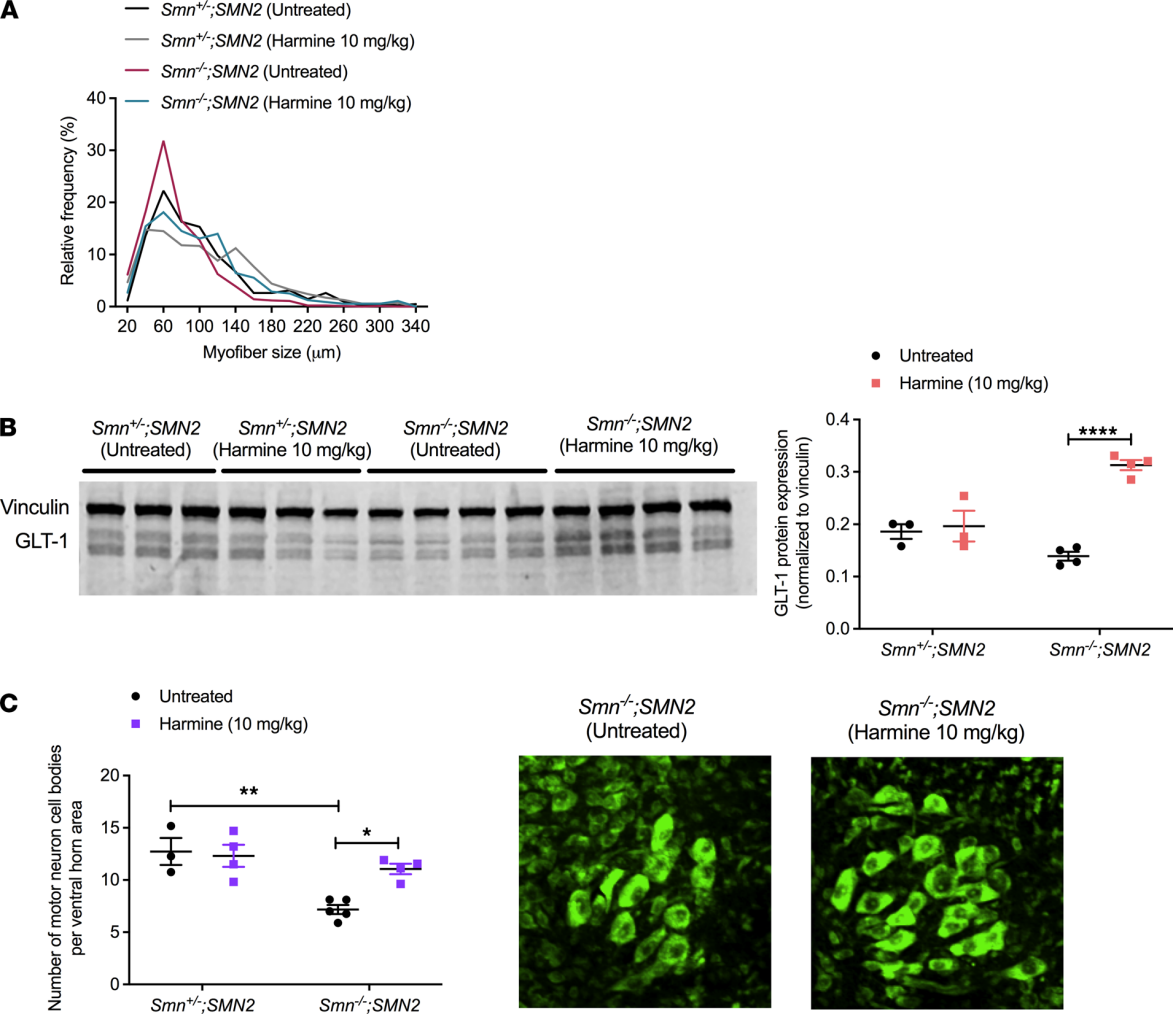
Administration of harmine to SMA mice improves neuromuscular phenotypes. All treated animals received a daily dose of harmine (10 mg/kg, diluted in 0.9% saline) by gavage starting at P0. (**A**) Relative frequency of myofiber sizes in P7 untreated and harmine-treated *Smn^–/–^;SMN2* and *Smn^+/–^;SMN2* mice. Data are shown as percentages, with *n =* 3 animals per experimental group and > 400 myofibers per experimental group. (**B**) Western blot and quantification of GLT-1/vinculin expression in the spinal cord of P7 untreated and harmine-treated *Smn^–/–^;SMN2* and *Smn^+/–^;SMN2* mice. Data are shown as a scatter plot and are represented as mean ± SEM; *n =* 3 for untreated and harmine-treated *Smn^+/–^;SMN2* mice, *n =* 4 for untreated and harmine-treated *Smn^–/–^;SMN2* mice, 2-way ANOVA followed by a Sidak’s multiple comparisons test, F = 35.01, df = 10, ****P < 0.0001. (**C**) Number of motor neuron cell bodies per ventral horn area in the spinal cord of P7 untreated and harmine-treated *Smn^–/–^;SMN2* and *Smn^+/–^;SMN2* mice. Data are represented as mean ± SEM *n =* 3 for untreated *Smn^+/–^;SMN2* mice, *n =* 4 for harmine-treated *Smn^–/–^;SMN2* and *Smn^+/–^;SMN2* mice, *n =* 5 for untreated *Smn^–/–^;SMN2* mice, 2-way ANOVA followed by a Tukey’s multiple comparisons test, F = 4.617, df = 12, *P < 0.05, **P < 0.01. Images are representative spinal cord ventral horn areas of untreated and harmine-treated *Smn^–/–^;SMN2* mice. Total original magnification, ×20.

Given that harmine was chosen to target molecular effectors in muscle, we measured the myofiber size in the TAs from P7 untreated and harmine-treated *Smn^–/–^;SMN2* and *Smn^+/–^;SMN2* mice. We observed an increased proportion of larger myofibers in harmine-treated *Smn^–/–^;SMN2* mice compared with untreated *Smn^–/–^;SMN2* animals (Figure 8A).

Harmine has also been reported to increase the expression of the neuroprotective glutamate transporter 1 (GLT-1; refs. 41, 42); thus, we assessed GLT-1 protein levels in P7 spinal cords from untreated and harmine-treated *Smn^–/–^;SMN2* and *Smn^+/–^;SMN2* mice. We found that harmine administration significantly increased GLT-1 expression in treated *Smn^–/–^;SMN2* mice compared with untreated animals, while having no impact in *Smn^+/–^;SMN2* healthy controls (Figure 8B and Supplemental Figure 5), suggesting an SMA-dependent effect.

Finally, given the reported neuroprotective activities of harmine (43), we proceeded to evaluate motor neuron loss in lumbar spinal cords of untreated and harmine-treated P7 *Smn^–/–^;SMN2* animals (Figure 8C). We observed that daily harmine administration significantly increased the number of motor neurons per ventral horn area in SMA mice, restoring it to the average number found in untreated and treated *Smn^+/–^;SMN2* healthy littermates (Figure 8C), further supporting a CNS-dependent effect of harmine.

We, thus, have demonstrated that treating SMA mice with harmine significantly improves multiple molecular and pathological phenotypes in both skeletal muscle and the spinal cord.

### Harmine administration restores gene expression in muscle of SMA mice.

**Figure 9:**
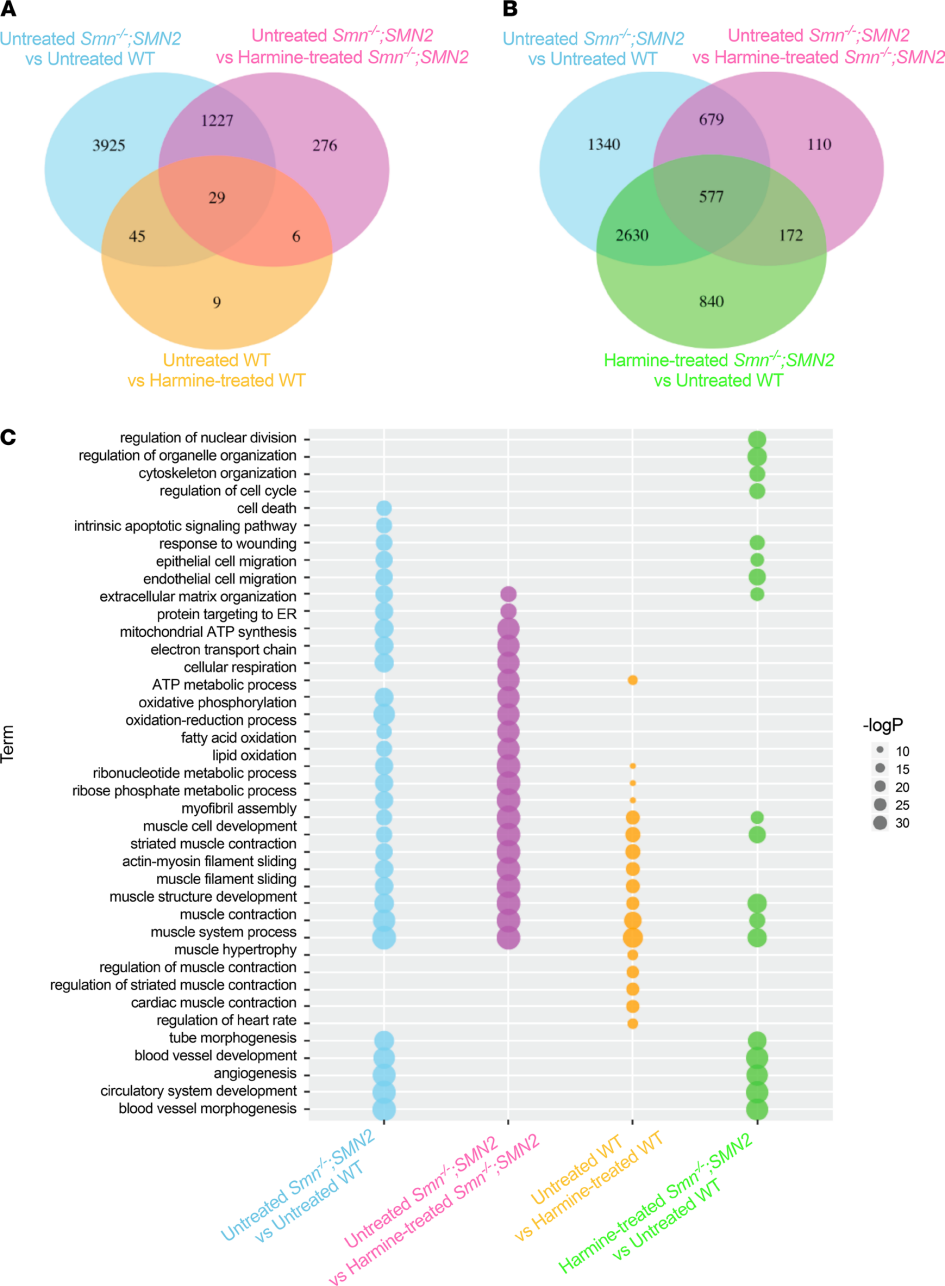
RNA sequencing and pathway analysis reveals full rescue of 20% of dysregulated genes in SMA muscle following harmine administration. All treated animals received a daily dose of harmine (10 mg/kg, diluted in 0.9% saline) by gavage starting at P0. TAs were harvested at P7 from untreated and harmine-treated *Smn^–/–^;SMN2* mice and WT animals and were processed for RNA sequencing. (**A**) Venn diagram representation of the differentially expressed (DE) genes based on the negative binomial distribution (DESeq2) in untreated *Smn^–/–^;SMN2* mice versus untreated WT mice (blue), harmine-treated *Smn^–/–^;SMN2* mice versus untreated *Smn^–/–^;SMN2* mice (purple), and untreated WT mice versus harmine-treated WT mice (orange). (**B**) Venn diagram representation of the DE genes based on the negative binomial distribution (DESeq2) in untreated *Smn^–/–^;SMN2* mice versus untreated WT mice (blue), harmine-treated *Smn^–/–^;SMN2* mice versus untreated *Smn^–/–^;SMN2* mice (purple), and harmine-treated *Smn^–/–^;SMN2* mice versus untreated WT mice (green). (**C**) Gene ontology (GO) biological processes enriched in genes DE in untreated *Smn^–/–^;SMN2* mice versus untreated WT mice (blue), in harmine-treated *Smn^–/–^;SMN2* mice versus untreated *Smn^–/–^;SMN2* mice (purple), in untreated WT mice versus harmine-treated WT mice (orange), and in harmine-treated *Smn^–/–^;SMN2* mice versus untreated WT (green). –LogP values for the enrichment are reported.

To systematically explore the effects of harmine in SMA muscle and further validate our combined bioinformatics and drug repurposing approach, we performed RNA sequencing (RNA-seq) on TAs from P7 untreated and harmine-treated *Smn^–/–^;SMN2* and WT mice. A total of 15,523 protein coding genes were identified across all samples. We found that harmine significantly reversed 1256 genes that are DE between *Smn^–/–^;SMN2* mice and WT animals (Figure 9A). Interestingly, harmine treatment in WT animals influenced the expression of substantially fewer genes than in *Smn^–/–^;SMN2* mice (Figure 9B), showing a high specificity toward pathways dysregulated in *Smn^–/–^;SMN2* mice, such as muscle phenotypes, lipid metabolism, and glucose metabolism (Figure 9C) (44–46). In agreement with the incomplete rescue of disease phenotypes in SMA mice, harmine treatment did not restore all DE genes (Figure 9B) or pathways (Figure 9C) such as muscle cell development and angiogenesis (47, 48). A complete list of enriched GO biological processes for the DE genes in each comparison is provided in Supplemental Table 3.

Considering the role of SMN in regulating RNA splicing (49), we examined whether harmine restored splicing alterations observed following loss of *Smn*. From a total of 81,011 distinct transcripts, 84 were found to be dysregulated in the disease model (*Smn^–/–^;SMN2* versus WT), of which only 1 was found to be reversed by harmine treatment — namely, DNA methyltransferase 3 β (*Dnmt3b*).

Thus, our RNA-seq analysis demonstrates that harmine reverses a large number of molecular pathologies in skeletal muscle of SMA mice beyond the selected candidate reporter genes, with a more prominent effect on overall expression than alternative splicing.

### Harmine restores multiple, but not all, molecular networks disturbed in muscle of Smn^–/–^;SMN2 mice.

**Figure 10:**
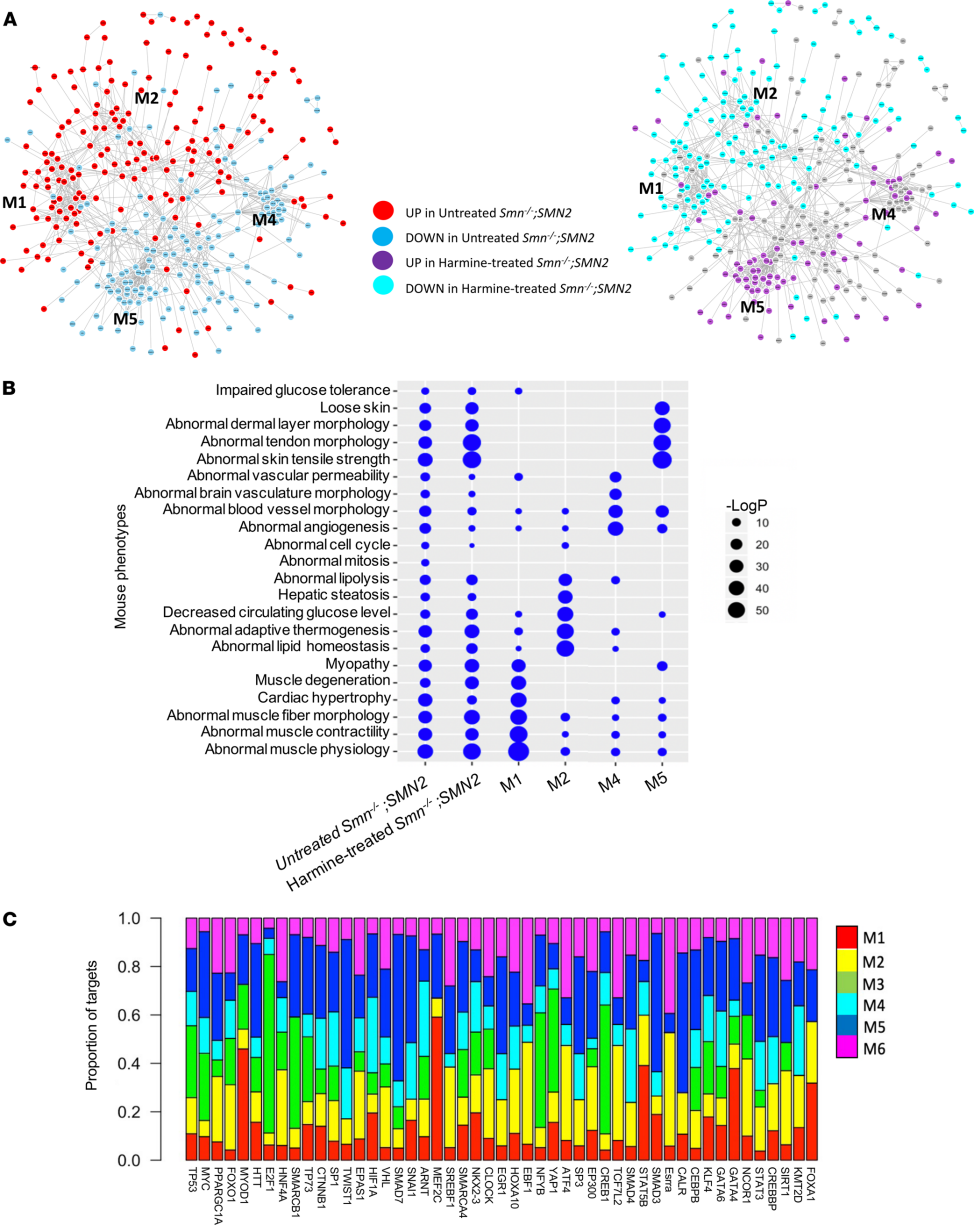
Identification of molecular effectors involved in harmine activity in SMA muscle. (**A**) A gene functional network was built extracting gene interactions from a Phenotypic Linkage Network (45) for the top 500 most differentially expressed (DE) genes (ordered by adjusted *P* value) in untreated *Smn^–/–^;SMN2* mice versus untreated WT mice. Genes are represented as nodes and are colored by direction expression change in untreated *Smn^–/–^;SMN2* mice versus untreated WT mice (left) and by direction of expression change in harmine-treated *Smn^–/–^;SMN2* mice versus untreated *Smn^–/–^;SMN2* mice (right). Gray nodes correspond to genes that are DE in the disease model (untreated *Smn^–/–^;SMN2* mice versus untreated WT) mice but have not been restored by harmine treatment. (**B**) Top MGI enriched phenotypes for the 4 identified modules in the network (shown in **A**) that show reversed expression profile after harmine treatment. –LogP values for the enrichment are reported. (**C**) Ingenuity Pathway Analysis (IPA) tool was used to identify upstream regulators of the top 500 most differentially expressed genes in untreated *Smn^–/–^;SMN2* mice versus untreated WT mice (shown in **A**). For each of the top 50 most significant upstream regulators shown (ordered on enrichment *P* values from left [most significant] to right [less significant]), we calculated the proportions of target genes within each of the 6 modules that are predicted to be regulated by the corresponding upstream regulator. Represented is a selected reduced list of regulators based on high proportion of target genes from Module 1 (muscle phenotypes) and Module 2 (glucose and lipid metabolism).

To further assess the restorative effects of harmine at a molecular level, we built a gene functional network from the top 500 DE genes using functional relationships defined by a phenotypic linkage network that links genes together that are likely to influence similar phenotypes (50). Louvain clustering of this network identified 6 modules of interconnected genes disturbed in muscle of *Smn^–/–^;SMN2* mice (Supplemental Figure 6), of which 3 (M1, M2, and M5) were fully restored and 1 (M4) was partially restored by harmine treatment (Figure 10A). Enrichment analysis in mouse phenotypes highlighted several pathways known to be involved in SMA, such as lipid and glucose metabolism (44, 46), as well as muscle fiber morphology and contraction (refs. 45, 47; Figure 10B), providing potential molecular explanations for the improved phenotypes in harmine-treated SMA mice and a similarity to the pathways associated with Pip6a-PMO treatment (Figure 2B). A tissue enrichment analysis on GTEx gene expression data confirmed the effect of harmine upon muscle-specific genes (Supplemental Figure 7). Through Ingenuity Pathway Analysis (IPA), we identified upstream regulators of the 6 modules of interconnected genes disturbed in muscle of *Smn^–/–^;SMN2* mice (Figure 10C). A complete list of upstream regulators and their downstream targets is provided in Supplemental Table 4.

Our large network analyses, therefore, suggest that additional mechanistic investigations of functional biological pathways are required to better understand the specific and direct benefits of harmine in SMA muscle. Importantly, our bioinformatic analyses have uncovered several interesting molecular networks restored by harmine in SMA muscle that could have further implications for future development of muscle-specific therapies for SMA.

## Discussion

Despite the tremendous recent advances in SMA gene therapy, this neuromuscular disorder remains incurable, and there is an urgent need for the development of second-generation treatments that can be used in combination with SMN-dependent therapies (16–18). In this study, we evaluated and validated a strategy combining transcriptomics, proteomics, and drug repositioning to identify therapeutic compounds that have the potential to improve muscle pathology in SMA. An in-depth investigation of one of these drugs, harmine, further supports our approach, since harmine restored several molecular, behavioral, and histological disease phenotypes in both cellular and animal models of the disease.

Of major importance, and to our surprise, we demonstrated that early SMN restoration via Pip6a-PMO corrects most, if not all, of the transcriptomic and proteomic dysregulations in SMA muscle, highlighting the need for and likely benefit from early treatment intervention in SMA. It is important to note, however, that the Pip6a-PMO dose delivered to mice was very high and most likely higher than what would be expected in patients. Our pathway analyses revealed that many molecular functions that are dysregulated in SMA mice compared with WT mice and recovered by Pip6a-PMO have previously been implicated in the pathology of SMA, including RNA metabolism and splicing, circadian regulation of gene expression, ubiquitin pathways, regulation of Rho protein signal transduction, and actin-binding pathways (51–54). Their normalization following SMN restoration further supports their involvement in SMA pathology.

Using the DE genes and proteins in SMA muscle compared with WT, we used a CMap perturbational data set to provide a list of candidate drugs that could improve SMA pathology, some of which had previously been evaluated in SMA, such as salbutamol (55). CMap analysis has been used to identify potential therapeutics for a range of different conditions, such as skeletal muscle atrophy (56), osteoarthritic pain (57), lung adenocarcinoma (58), and kidney disease (59). CMap can also help establish prediction models for different adverse drug reactions and evaluate drug safety (60).

In this study, we chose to provide a more in-depth assessment of harmine, a drug predicted to restore DE genes and proteins in SMA muscle. Harmine is a β-carboline alkaloid and has various vasorelaxant, antiinflammatory, antimicrobial, analgesic, antioxidative, antimutagenic, antitumor, antidepressive, antiaddictive, and neuroprotective therapeutic effects (33, 61, 62). The pharmacological mechanisms involve several molecular targets including monoamine oxidase (MAO), serotonin 5-HT2A/C receptors, imidazoline I1/2 receptors, reactive oxygen species (ROS), dual specificity tyrosine phosphorylation–regulated kinase 1A (DYRK1A), GLT-1, and neurotrophic factors (33, 61, 62). In our study, one of the genes downregulated in SMA muscle compared with WT animals and increased by harmine was *Snrnp27*, a small nuclear RNP (snRNP) involved in pre-mRNA splicing (63), and SMN plays a canonical role in the assembly of snRNPs (64). Of note, while the observed change in *Snrnp27* levels were small and further investigations are required to fully determine its biological significance, it was nevertheless observed in both SMA mice and healthy littermates, suggesting a potential direct and beneficial effect of harmine administration on *Snrnp27* expression. *Cdkn1a* (or *p21*) was also identified as a potential molecular target of harmine. This mediator of cell cycle and DNA repair is reported to be upregulated in various SMA models (65–69). While we validated an upregulated expression of *Cdkn1a* in skeletal muscle of symptomatic SMA mice, harmine administration did not lead to its predicted downregulation in vivo. Moreover, in our in vitro experiments, harmine actually increased *Cdkn1a* expression in certain cell types and at certain doses, further highlighting the importance of validating in situ predictions in relevant cell and animal models. Indeed, harmine did not demonstrate a predicted activity on all selected candidate reporter genes, and any observed activity varied between cell types and tissues. Given that the CMap analysis is primarily based on data from human cancer cell lines (MCF7, PC3, and HL60), distinct effects across cell types and tissues are to be expected. While harmine influenced a subset of the selected candidate reporter genes in the predicted direction, our RNA-seq analysis demonstrated that harmine does, in fact, normalize the expression of a large number of additional genes in skeletal muscle of SMA mice that are implicated in key muscle processes such as muscle structure development, muscle contraction, muscle system process, and muscle cell differentiation. Thus, our combined transcriptomics, proteomics, and CMap approach has not only identified genes that have previously been implicated in SMA pathology, but has also provided an extensive list of potentially novel and relevant molecular targets for further mechanistic investigations and therapeutic development.

Harmine can cross the blood-brain barrier and has well-characterized neuroprotective properties, including its ability to upregulate the expression of GLT-1 in several neurodegenerative models (41, 42). We indeed showed that GLT-1 expression is significantly upregulated in the spinal cord of SMA mice following harmine administration, which could potentially counteract the reduced glutamate transporter activity that has previously been reported throughout the CNS of SMA patients (70). In addition, we found that harmine significantly increased the number of motor neurons in the spinal cord of SMA animals. However, it is unclear whether this prevention of motor neuron loss is a cause or a consequence of the improved weight and lifespan, simply reflects a delayed neurodegenerative process, and/or is associated with functional improvements. Given that the extent of motor neuron loss is quite similar between SMA mouse models of varying severities, motor neuron health and function are most likely better correlated with disease progression than absolute number (71). Nevertheless, the fact that harmine exerted muscle and CNS effects makes it an interesting therapeutic option for SMA. However, it is important to note that harmine can also exert adverse effects such as the onset of tremors (38–40), which we observed when dosing the intermediate *Smn^2B/–^* mouse model over a longer period of time.

Notably, the diverse phenotypic changes observed in SMA mice occurred in spite of harmine’s short half-life of 1–3 hours (72), suggesting that the observed restoration of gene networks was sustained either through regulatory cascades and/or a self-reinforcement. Performing time-series or pseudotemporal analyses of the responding regulatory gene networks could elucidate the key reinforcing drivers. Although SMN protein levels were not increased and harmine treatment did not rescue the entire perturbed gene networks, the specificity of harmine treatment in skeletal muscle is remarkable, with very few affected genes outside of the perturbed gene networks. It is important to also consider that the benefits of harmine in SMA mice may be due to direct effects in the target muscle tissue and/or indirect effects via improved phenotypes in the spinal cord and in additional pathologically affected peripheral tissues (e.g., heart, liver, pancreas; ref. 73) previously demonstrated to be functionally modulated by harmine (74–76) and not evaluated in the current study. Thus, while harmine itself might not be the ideal SMA treatment due to its range of pharmacological and adverse side effects (77), replicating harmine’s tissue-specific activities with more targeted compounds may prove an effective strategy for SMA therapeutic development.

To our knowledge, this is the first in-depth validation of this combinatorial approach in SMA. We were able to show the strength and potential of combining multiomics and drug repositioning to uncover potentially novel therapeutic entities, which in this case was aimed at improving muscle health in SMA. Our work, thus, provides an invaluable list of pharmacological compounds, upstream regulators, and molecular targets that can be evaluated for treatment of SMA muscle pathology, as well as strong support for the use of this combined multiomics and bioinformatic strategy.

## Methods

### Animals and animal procedures.

WW mice (FVB/N and C57BL/6J; refs. 78, 79) were obtained from the Jackson Laboratory. The severe *Smn^–/–^;SMN2^+/–^* mouse model (22) was also obtained from The Jackson Laboratory (FVB.Cg-*Smn1tm1Hung Tg[SMN2]2Hung/J*). The moderate *Smn^2B/–^* mouse model (27, 80) was provided by Lyndsay M. Murray (Centre for Discovery Brain Sciences, University of Edinburgh, Edinburgh, United Kingdom). All experiments with live animals were performed at the Biomedical Services Building, University of Oxford. For all experiments, litters were randomly assigned at birth and whole litters composed of both sexes were used. Sample sizes were determined based on similar studies with SMA mice. For survival curves, the following humane endpoints, as defined in our Home Office Project Licence, were used. (a) For the *Smn*^–/–^*;SMN2* mice, animals were killed when they demonstrated either of the following clinical signs: hindlimb paralysis, immobility, inability to right (greater than 30 seconds), and greater than 15% weight loss. (b) For the *Smn^2B/–^* mice, animals were killed when they demonstrated either of the following clinical signs: hindlimb paralysis, immobility, inability to right (greater than 30 seconds), and greater than 18% weight loss.

The Pip6a-PMO and Pip6a-scrambled conjugates were both separately prepared in 0.9% saline solution and administered at a dose of 10 μg/g via an i.v. facial vein injection at P0 and P2.

Harmine hydrochloride (sc-295136, Insight Biotechnology Ltd., Santa Cruz Biotechnology Inc.) was dissolved in 0.9% saline and administered daily (10 mg/kg) by gavage.

### Synthesis of Pip6a peptide-PMO conjugates.

The PMO sequence targeting ISS-N1 intron 7 (–10, –27) (5′-ATTCACTTTCATAATGCTGG-3′) and scrambled PMO (5′-TAC GTT ATA TCT CGT GAT AC-3′) were purchased from Gene Tools LLC (Corvallis).

The Pip6a Ac-(RXRRBRRXRYQFLIRXRBRXRB)-COOH peptide was manufactured by standard 9-fluorenylmethoxy carbonyl chemistry, purified to > 90% purity by reverse-phase high-performance LC (HPLC) and conjugated to the 3′ end of the PMO through an amide linkage. The conjugate was purified by cation exchange HPLC, desalted, and analyzed by MS. Pip6a peptide-PMO conjugates were dissolved in sterile water and filtered through a 0.22 μm cellulose acetate membrane before use.

### Laminin staining of skeletal muscle.

TA muscles were fixed in 4% PFA overnight. Tissues were sectioned (13 μm) and incubated in blocking buffer for 2 hours (0.3% Triton-X, 20% FBS, and 20% normal goat serum in PBS; all from MilliporeSigma). After blocking, tissues were stained overnight at 4°C with rat anti-laminin (1:1000, L0663, Sigma-Aldrich) in blocking buffer. The next day, tissues were washed in PBS and probed using goat anti–rat IgG 488 secondary antibodies (1:500, AlexaFluor 488, Thermo Fisher Scientific) for 1 hour. PBS-washed tissues were mounted in Fluoromount-G (Southern Biotech). Images were taken with a DM IRB microscope (Leica). Quantitative assays were performed in a blinded fashion on 3–5 mice for each group and 5 sections per mouse. The area of muscle fiber within designated regions of the TA muscle sections was measured using Fiji (81).

### Nissl staining of spinal cord.

Whole spinal cords were fixed in 4% PFA overnight and subsequently placed in a 30% sucrose solution (PBS). The lumbar areas of the spinal cords were then flash-frozen in a 50:50 mixture of OCT compound/30% sucrose, and 20 μm sections were cut. Sections were first rehydrated 40 minutes in PBS followed by a 10-minute permeabilization step in 0.1% Triton-X. Sections were washed in PBS and stained with Neurotrace 500/525 green fluorescent Nissl (1:500, N21480, Thermo Fisher Scientific). Sections were then washed in PBS, counterstained with DAPI, and mounted in Fluoromount-G (Southern Biotech). Images for quantification were taken with a DM IRB microscope (Leica). Motor neuron cell body counts in the ventral horn area of the spinal cord were performed blindly on 3–5 mice per experimental group and 5 sections per mouse using Fiji (81). Representative images were taken with an Olympus Fluoview FV1000 confocal microscope and processed with Fiji (81).

### qPCR.

RNA was extracted from tissues and cells by either a RNeasy kit from Qiagen or by guanidinium thiocyantate-acid-phenol-chloroform extraction using TRIzol Reagent (Invitrogen) as per manufacturer’s instructions. The same RNA extraction method was employed for similar experiments, and equal RNA amounts were used between samples within the same experiments. cDNA was prepared with the High Capacity cDNA Kit (Invitrogen) according to the manufacturer’s instructions. The cDNA template was amplified on a StepOnePlus Real-Time PCR Thermocycler (Invitrogen) with SYBR Green Mastermix from Applied Biosystems. qPCR data were analyzed using the StepOne Software v2.3 (Applied Biosystems). Primers used for qPCR were obtained from IDT, and sequences for primers were either self-designed or ready-made (Supplemental Table 5). Relative gene expression was quantified using the Pfaffl method (82), and primer efficiencies were calculated with the LinRegPCR software. We normalized the relative expression level of all tested genes in mouse tissue and cells to *RNA polymerase II polypeptide J* (*PolJ*) (83). For human cells, we ran a GeNorm kit (Primer Design) to identify ribosomal protein L13a (*RPL13A*) as a reference/housekeeping gene. Primers for *RPL13A* were from IDT (Assay ID, Hs.PT.58.47294843).

### Cell culture.

Both C2C12 (ATCC, CRL-1772; ref. 84) and NSC-34 (provided by Peter Claus, Hannover Medical School, Hannover, Germany; ref. 85) cell lines were maintained in growth media consisting of DMEM supplemented with 10% FBS and 1% penicillin/streptomycin (all from Invitrogen). The cells were cultured at 37°C with 5% CO_2_. C2C12 myoblasts were differentiated in DMEM containing 2% horse serum (HS) for 7 days to form multinucleated myotubes.

Human fibroblasts were obtained from Coriell Institute (SMA GM03813, control AG02261) and cultured in DMEM, supplemented with 1% antibiotics/antimycotics and 20% FBS.

### MTS assays.

Cell viability and proliferation of C2C12 and NSC-34 cells treated with harmine (sc-202644, Insight Biotechnology Ltd., Sante Cruz Biotechnology Inc.) dissolved in DMSO (final concentration 0.03%) were evaluated with a 3-(4,5-dimethylthiazol-2-yl)-5-(3-carboxymethoxyphenyl)-2-(4-sulfophenyl)-2H-tetrazolium (MTS) assay kit (Colorimetric). The measurements were made according to manufacturer’s instructions. Briefly, 10 μL of MTS reagent was added directly to the wells, and cell plates were incubated at 37°C for a minimum of 1 hour. Absorbance was measured at 490 nm on a CLARIOstar plate reader (BMG LABTECH). Background absorbance was first subtracted using a set of wells containing medium only, and it was then normalized to and expressed as a relative percentage of the plate-averaged untreated control. To chemically induce apoptosis, cells were treated with 10 μM Staurosporine (Abcam).

### Western blot.

Freshly prepared radioimmunoprecipitation (RIPA) buffer was used to homogenize tissue and cells, consisting of 50 mM Tris (pH 8.8), 150mM NaCl, 1% NP-40, 0.5% Sodium Deoxycholate, 0.1% SDS, and complete mini-proteinase inhibitors (1 tablet per 10 mL extraction solution; Roche). Equal amounts of total protein were loaded, as measured by Bradford Assay. Protein samples were first diluted 1:1 with Laemmli sample buffer (Bio-Rad) containing 5% β-mercaptoethanol (MilliporeSigma) and heated at 100°C for 10 minutes. Next, samples were loaded on freshly made 1.5 mm 12% polyacrylamide separating and 5% stacking gel, and electrophoresis was performed at 120 V for approximately 1.5 hours in running buffer. Subsequently, proteins were transferred from the gel onto to a polyvinylidene fluoride (PVDF) membrane (MilliporeSigma) via electroblotting at 120 V for 60 minutes in transfer buffer containing 20% methanol. Membranes were then incubated for 2 hours in Odyssey Blocking Buffer (Licor). The membrane was then probed overnight at 4°C with primary antibodies (rabbit anti–GLT-1, 1:1000, Abcam, ab41621; mouse anti-vinculin, 1:200,000, Sigma-Aldrich, V9131) in Odyssey Blocking Buffer and 0.1% Tween-20. The next day, after three 10-minute washing steps with PBS, the membrane was incubated for 1 hour at room temperature with secondary antibodies conjugated to infrared dyes (goat anti–rabbit IgG [H + L], IRDye 800CW, LI-COR Biosciences, 827-08365; goat anti–mouse IgG [H + L], IRDye 680RD, LI-COR Biosciences, 926-68070). Lastly, the membrane was washed again 3 times for 10 minutes in PBS and visualized by scanning 700 nm and 800 nm channels on the LI-COR Odyssey CLx infrared imaging system (LI-COR) for 2.5 minutes per channel. The background was subtracted, and the signal of protein of interest was divided by signal of the housekeeping protein or total protein, per sample.

### Proteomic analysis.

Proteomic analyses were performed using a LC-MS–based method. High-resolution isoelectric focusing (HiRIEF) was used at the peptide level in the 3.7–5.0 pH range. Two tandem mass tags (TMTs, chemical labels) were used for MS-based quantification and identification of proteins. The data were median normalized based on peptide ratio. Among a total of 9798 potentially detectable proteins, most (8152 proteins) were identified in all samples/groups.

The limma R package was used for differential expression analysis, whereby DE proteins were defined by FDR < 0.05. GO enrichment analysis of proteomic data were executed using topGO R function, and adjusted *P* values were found for multiple testing following a Benjamini-Hochberg correction. For PCA, we used the prcomp R function on the normalized expression data.

### Microarray analysis.

RNA was extracted by guanidinium thiocyantate-acid-phenol-chloroform extraction using TRIzol Reagent (Invitrogen) as per manufacturer’s instructions. GeneChip Mouse Transcriptome Assay 1.0 arrays were used (Affymetrix core facility, Karolinska Institute) with 100 ng of RNA per sample. Annotations for the Mouse Transcript Array 1.0 at the transcript level were obtained from the Affymetrix website (http://www.affymetrix.com/products_services/arrays/specific/mo_trans_assay.affx#1_4). We performed background correction and RMA normalization at the probe level using oligo R package. We summarized the data in ensemble transcript IDs using the average. The total number of ensemble transcript IDs was 93,594, corresponding to 37,450 genes. For differential expression analysis, we used the limma R package and considered a transcript DE if their FDR < 0.05. A gene was considered DE if at least 1 of the associated transcripts was DE. GO enrichment analysis was performed in R using the topGO function as described for proteomic data. For PCA, we used the prcomp R function on the RMA normalized gene expression data at the gene level (for comparison with proteomic data).

### Combined analysis of proteomic and transcriptomic data.

To measure the similarity between gene expression profiles, we used the Ward hierarchical clustering on the Euclidean distance of 1 – *r* (where *r* is the Pearson correlation between samples). To compare the 2 omics readouts, proteomic and transcriptomic data were scaled (transformed to *Z*-score values), followed by a PCA showing that PC1 divides the data at the transcript and protein levels. Using the kill.pc function in the swamp R package, we extracted a new expression matrix where the variance given by PC1 had been removed. Finally, we performed hierarchical clustering analysis on the new expression matrix.

### RNA-seq analysis.

RNA was extracted using a RNeasy Microarray Tissue Mini Kit from Qiagen. Lysis and homogenization were performed using QIAzol Lysis Reagent. cDNA synthesis and RNA-seq library construction were performed at the Oxford Genomics Centre using poly(A) enrichment of the mRNA (mRNA-seq) and HiSeq 4000 Systems for sequencing. All samples passed quality control. For differential expression analysis, we used DESeq2 on genes expressed across all samples (15,523 genes) after removal of 1 outlier (harmine-treated *Smn^–/–^;SMN2* sample 1). We considered a gene DE at FDR < 0.05. For GO enrichment analysis, we used topGO R function and adjusted *P* values for multiple testing following a Benjamini-Hochberg correction. For mouse phenotype enrichment analysis, we downloaded phenotypes from the Mouse Genome Database, Mouse Genome Informatics, The Jackson Laboratory (http://www.informatics.jax.org) and used an in-house script to correct for the background set of expressed genes.

### Differential isoform expression analysis.

Transcript counts were first obtained using Salmon software v.0.11.2 (86). Differential isoform usage was then analyzed using edgeR package (87), considering an isoform as DE when the adjusted *P* value in the comparison between samples was below 0.05.

### Gene functional network and clustering method.

A gene functional network was built by extracting interactions from a phenotypic linkage network (50) for the top 500 most DE genes in *Smn^–/–^;SMN2* mice versus WT mice. To identify modules of highly interconnected genes in the network, we employed “cluster_louvain” function in “igraph” R package (88). This function implements the multilevel modularity optimization algorithm (89, 90) where, at each step, genes are reassigned to modules in a greedy way, and the process stops when the modularity does not increase in a successive step.

### Upstream regulators.

IPA (QIAGEN; www.qiagenbioinformatics.com) was used to identify the top 50 upstream regulators for the top 500 most DE genes in *Smn^–/–^;SMN2* mice versus WT mice. A reduced list of regulators was identified based on enrichment of their target genes within the 4 modules in the network that are restored upon harmine treatment.

### GTEx tissue enrichment analysis.

GTEx V7 tissue gene expression profiles were downloaded from gtexportal.org. For each tissue, we averaged the gene expression profiles across individuals, and we then identified tissue-specific genes as those with a fold change > +5 calculated for the expression in one tissue compared with all other tissues. Gene enrichment *P* values (hypergeometric test) were computed for the overlap between the identified tissue-specific gene sets and our sets of DE genes.

### CMap analysis.

Ensembl transcript identifications (IDs) from mice were mapped to human probe IDs (HG-U133A) using biomaRt (Ensembl transcript ID *mus musculus* → Ensembl gene ID *mus musculus* → ortholog_one2one → Ensembl gene ID *homo sapiens* → HG-U133A ID). We compared the identified disease and Pip6a-PMO signatures (top 500 upregulated and top 500 downregulated transcripts/proteins) to 6100 drug instances contained at CMap (Build 02, https://www.broadinstitute.org/connectivity-map-cmap). Each instance corresponds to a drug response (treatment versus vehicle control) in a particular cell line and covers up to 1230 drugs across mainly 3 human cell lines (MCF7 = 3095 instances, PC3 = 1741 instances, HL60 = 1229 instances, ssMCF7 = 18 instances, and SKMEL5 = 17 instances). We used the proven CMap algorithm; however, it is important to note that, although some improvements have been proposed, they have not been systematically evaluated (91). Briefly, each subset of up- and downregulated genes is compared with each instance by taking into account the ranked differences using a nonparametric rank test (Kolmogorov-Smirnov statistic). For each instance, a connectivity score (ranging from +1 to –1) represents the relative strength in which a drug induced (+ 1) or reversed (–1) a given gene signature, while zero indicates a random distribution of up- and downregulated genes in the ranked response of a drug.

### Data availability.

The data sets generated during and/or analyzed during the current study are included in this published article (and its supplementary material).

The expression data discussed in this publication have been deposited in NCBI’s Gene Expression Omnibus (92) and are accessible through GEO Series accession no. GSE150510 (https://www.ncbi.nlm.nih.gov/geo/query/acc.cgi?acc=GSE150510) and GSE150517 (https://www.ncbi.nlm.nih.gov/geo/query/acc.cgi?acc=GSE150517) for RNA-Seq and microarray data, respectively.

Associated raw data for the proteomics analysis can be found in the Proteomics Source Data file.

### Statistics.

All nonbioinformatic statistical analyses were done with the most Graphpad Prism software (version 8.4.2). When appropriate, a Student’s unpaired 2-tailed *t*-test, a 1-way ANOVA, or a 2-way ANOVA was used. Post hoc analyses used are specified in figure legends. Outliers were identified via the Grubbs’ test. For the Kaplan-Meier survival analysis, the log-rank test was used, and survival curves were considered significantly different at *P* < 0.05.

### Study approval.

Experimental procedures were authorized and approved by the University of Oxford ethics committee and UK Home Office (current project license PDFEDC6F0, previous project license 30/2907) in accordance with the Animals (Scientific Procedures) Act 1986.

## Author contributions

Conceptualization was contributed by MJAW, CW, and MB. Methodology was contributed by KEM, VV, JMS, CW, and MB. Software was contributed by VV, JMS, and CW. Validation was contributed by KEM, VV, JMS, CW, and MB. Formal analysis was contributed by KEM, VV, JMS, JMH, CW, and MB. Investigation was contributed by KEM, VV, JMS, JMH, SMH, OGDJ, GH, NA, and MB. Resources were contributed by FA. Data curation was contributed by VV, JMS, and CW. Original draft preparation was contributed by KEM, VV, JMS, CW, and MB. Review and editing of the manuscript was contributed by KEM, VV, JMS, JMH, SMH, FA, OGDJ, GH, NA, MJAW, CW, and MB. Visualization was contributed by KEM, VV, JMS, and MB. Supervision was contributed by MJAW, CW, and MB. Project administration was contributed by CW and MB. Funding acquisition was contributed by SMH, MJAW, CW, and MB.

## Supplementary Material

Supplemental data

Supplemental Data Set 1

Supplemental Table 1

Supplemental Table 2

Supplemental Table 3

Supplemental Table 4

Supplemental Table 5

## Figures and Tables

**Table 1:**
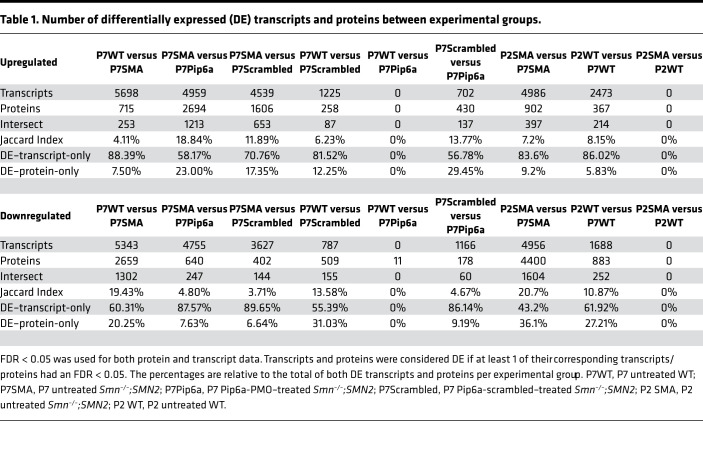
Number of differentially expressed (DE) transcripts and proteins between experimental groups.

**Table 2:**
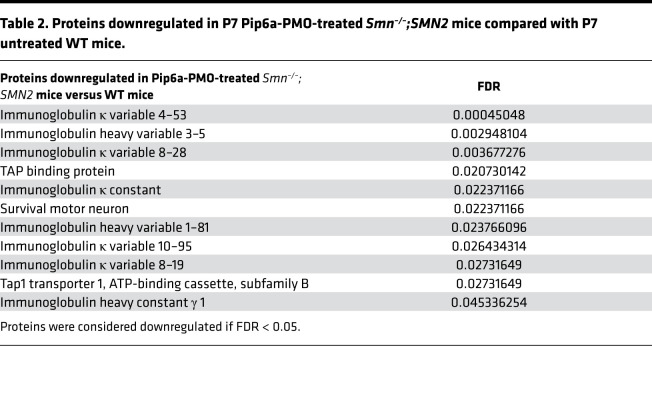
Proteins downregulated in P7 Pip6a-PMO-treated *Smn^–/–^;SMN2* mice compared with P7 untreated WT mice.

**Table 3:**
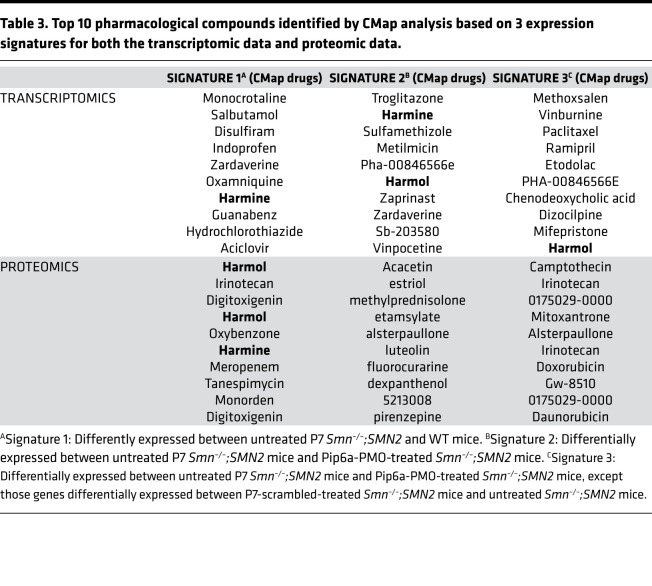
Top 10 pharmacological compounds identified by CMap analysis based on 3 expression signatures for both the transcriptomic data and proteomic data.

## Version 1 07/08/2021

Electronic publication
