# Supplementary material for: Combining multiomics and drug perturbation profiles to identify muscle-specific treatments for spinal muscular atrophy
Source: JCI Insight. 2021 Jul 8;6(13):e149446. doi: 10.1172/jci.insight.149446 (PMC8410072; doi:10.1172/jci.insight.149446)
Supplement: Supplemental data [file jciinsight-6-149446-s219.pdf]

SUPPLEMENTAL FIGURES

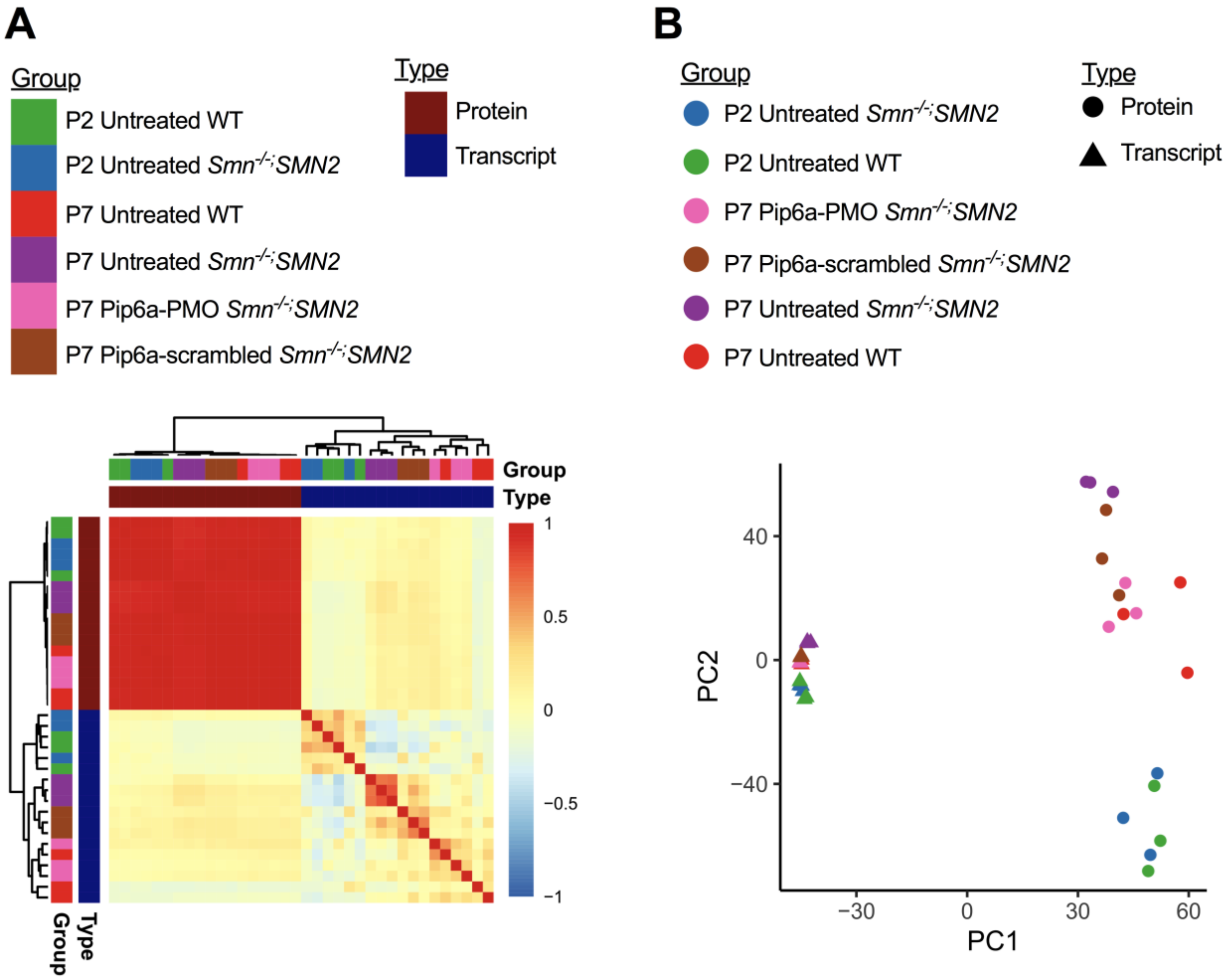

Supplementary Figure 1. Clustering and principal component analysis integrating proteomic and transcriptomic data. (**A**) Heatmap corresponds to the Pearson correlation between each pair of proteomic and transcriptomic profiles. (B) Principal component analysis of the proteomic and transcriptomic data separated by experimental group and by type (transcripts or protein) along the first two components.

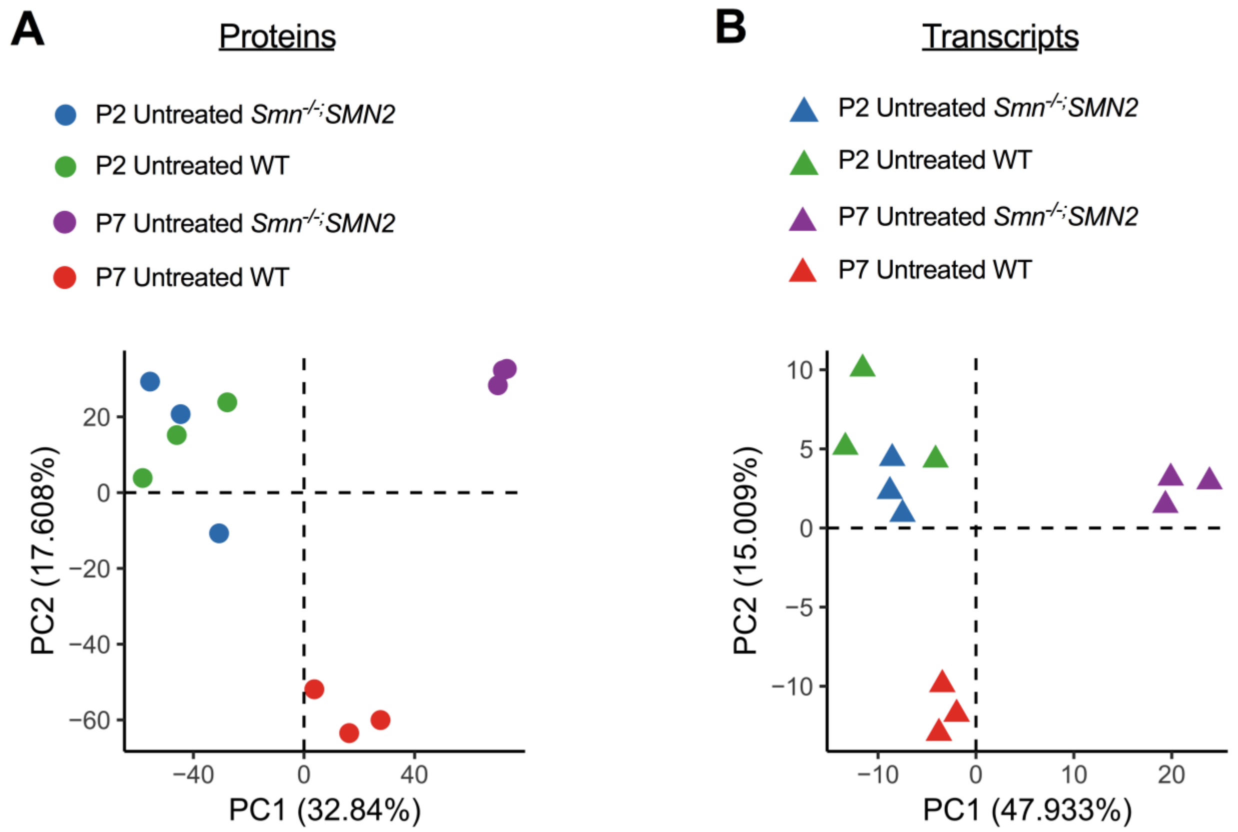

Supplemental Figure 2. Principal component analysis on proteomic (A) and transcriptomic (B) data of post-natal day (P) 2 and P7 untreated *Smn$^{-/-}$;SMN2* and WT mice.

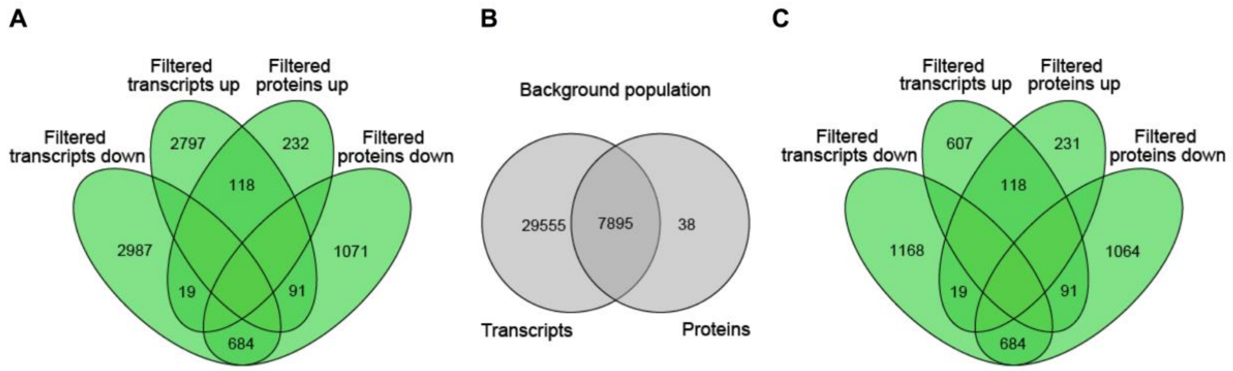

Supplemental Figure 3. Overlap between filtered gene signatures at the transcript and protein level. (A) Venn diagram indicating the number of overlapping and unique genes at the transcript and protein levels for the filtered sets of differentially expressed genes (DEGs) (as indicated in Figure 2). (**B**) Venn diagram indicating the overlap between the gene background populations measured at the transcript and protein level. (**C**) Venn diagram indicating the overlap between the filtered gene signatures at the transcript and protein level that are contained in the shared background population of 7895 genes.

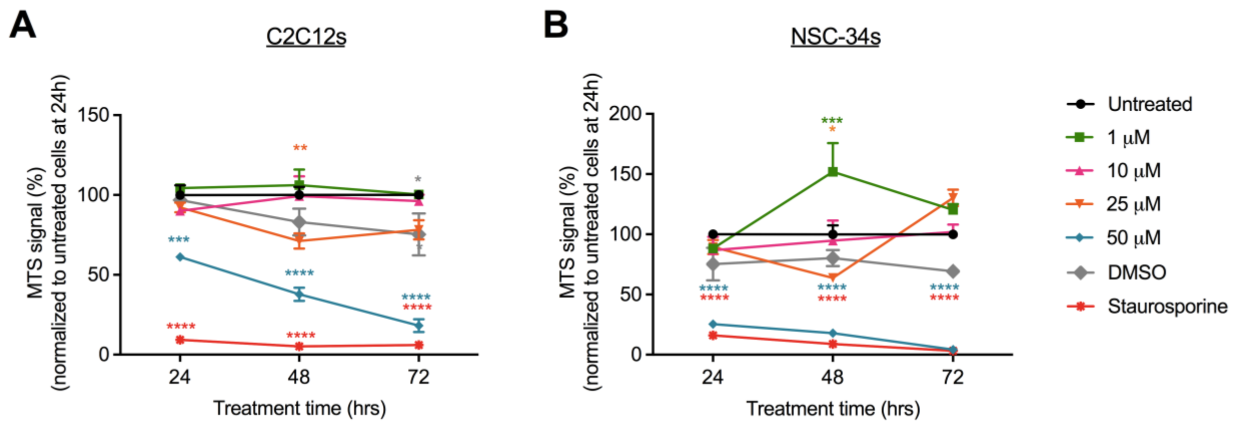

Supplemental Figure 4. *In vitro* Dose-dependent toxicity of harmine treatment *in vitro*. C2C12s (**A**) and NSC-34s (**B**) were treated with 1, 10, 25 or 50 µM for 24, 48 or 72 hours. Control groups were untreated cells or cells treated with either DMSO (vehicle) or Staurosporine (positive control). An MTS assay was performed on all experimental groups and MTS scores are normalized to untreated cells at 24 hours (100%). Data are mean ± SEM, n = 3 independent wells, two-way ANOVA followed by a Dunnett's multiple comparisons test, F = 116.2 (C2C12s), F = 82.04 (NSC-34s), df = 42 for all, *p<0.05, ***p*<0.01, ****p*<0.001, *****p*<0.0001.

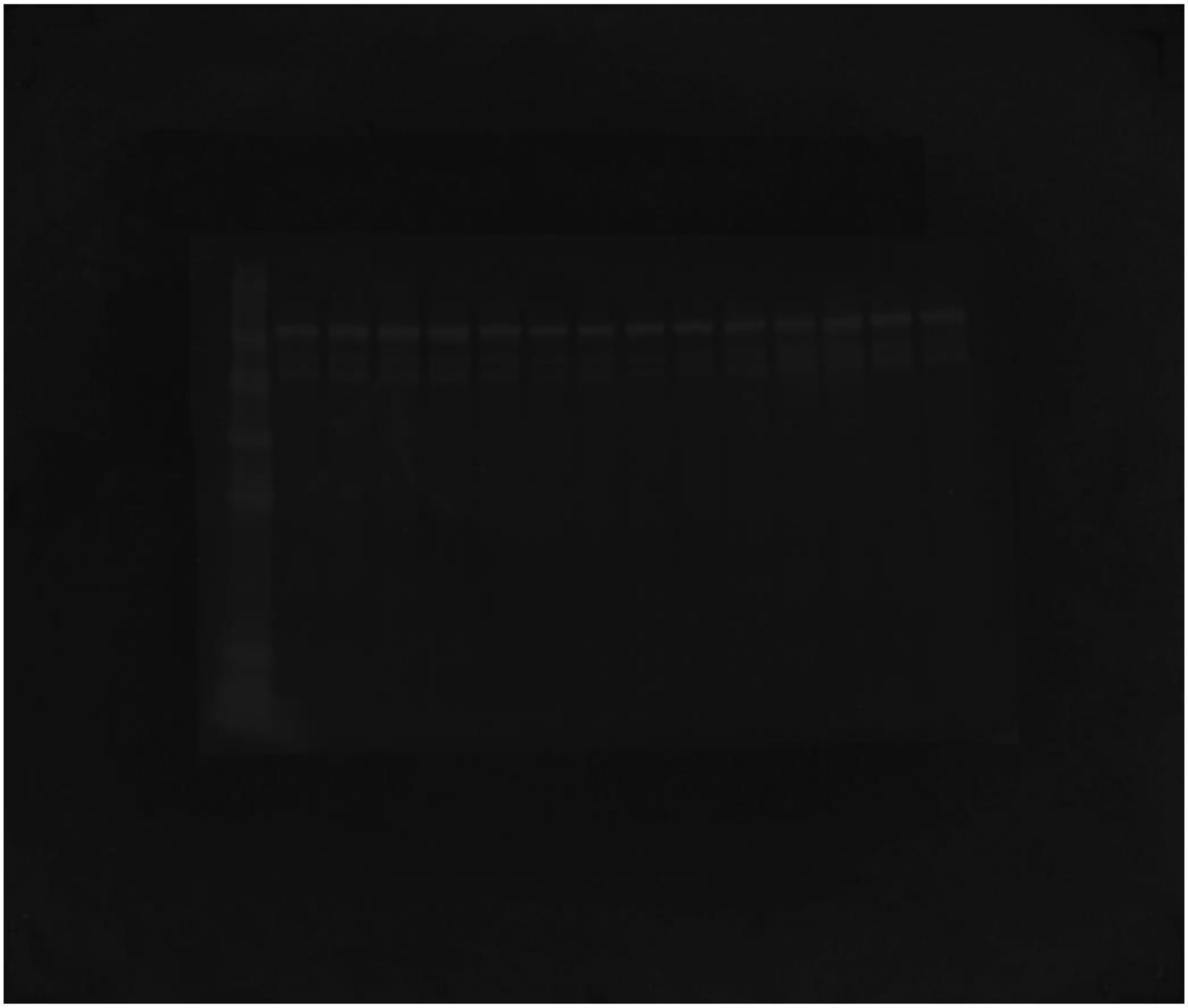

Supplemental Figure 5. Unprocessed raw image of vinculin and GLT-1 immunoblot.

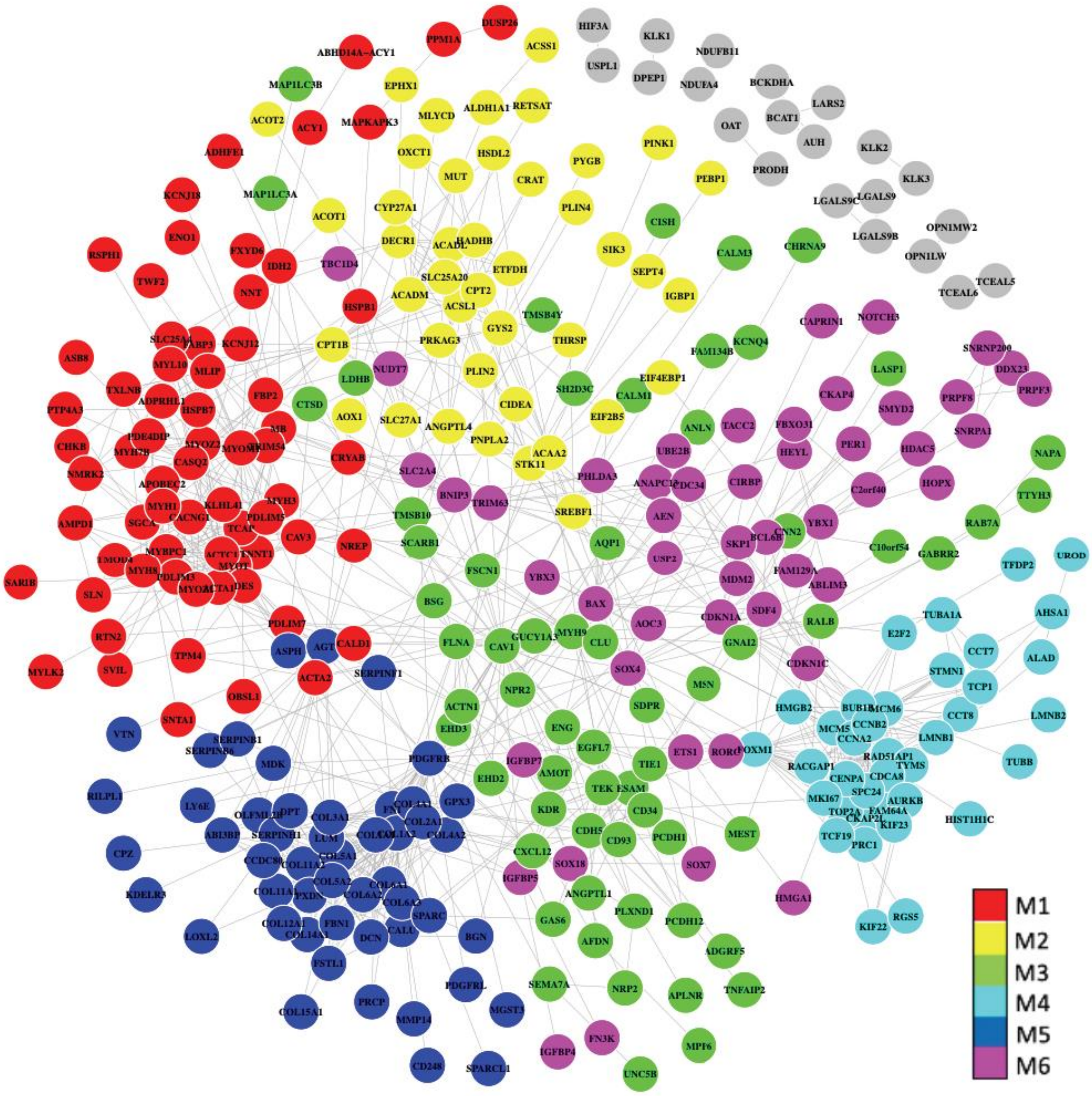

Supplemental Figure 6. A gene functional network for the top 500 most differentially expressed genes in untreated *Smn*$^{-/-}$*;SMN2* mice vs untreated WT mice. Genes are represented as nodes and are coloured by module identified through Louvain clustering.

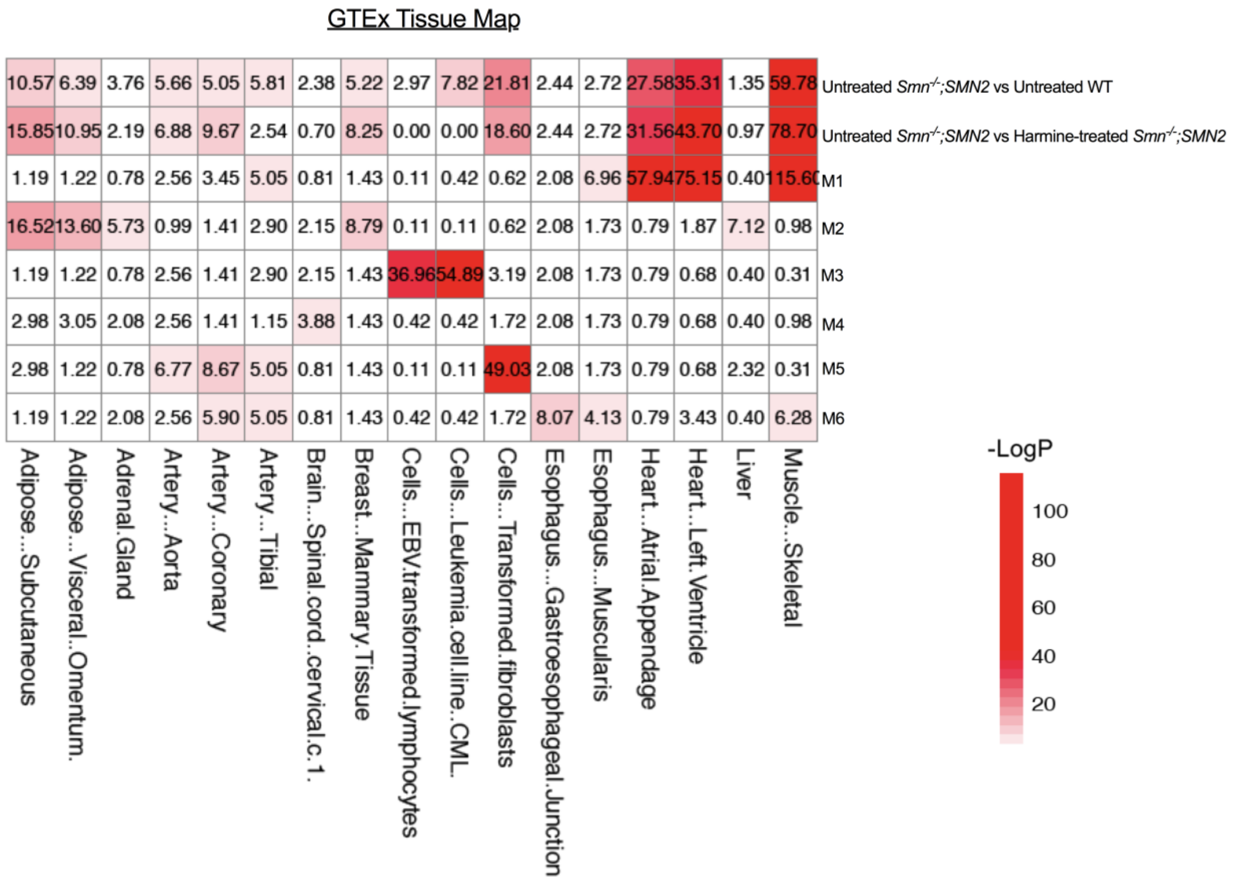

Supplemental Figure 7. GTEx-tissue enrichment analysis for differentially expressed (DE) genes shows harmine significantly alters muscle-specific genes, and more specifically, gene modules associated with muscle and adipose tissue-associated gene expression. For each GTEx tissue, we identified tissue-specific genes as those with a fold change >+5 calculated from the expression in one tissue compared to all other tissues. Gene enrichment *p*-values (hypergeometric test) were computed for the overlap between the identified tissue-specific gene sets and our sets of differentially expressed genes in untreated *Smn*$^{-/-}$*;SMN2* mice vs untreated WT mice, in untreated *Smn*$^{-/-}$*;SMN2* mice vs harmine-treated *Smn*$^{-/-}$*;SMN2* mice and in the six identified modules of highly interconnected genes in the network. The figure shows -log(p-values) for the enrichment in those tissues that reached significance (*p*-value<0.05) in at least one of the comparisons.
