# Supplementary material for: Combining multiomics and drug perturbation profiles to identify muscle-specific treatments for spinal muscular atrophy
Source: JCI Insight. 2021 Jul 8;6(13):e149446. doi: 10.1172/jci.insight.149446 (PMC8410072; doi:10.1172/jci.insight.149446)
Supplement: Supplemental Table 5 [file jciinsight-6-149446-s225.pdf]

SUPPLEMENTAL TABLES

Supplemental Table 1. List of transcripts and proteins differentially expressed between Pip6a-scrambled-treated *Smn*$^{-/-}$;*SMN2* mice vs untreated WT (FDR < 0.05).

Supplemental Table 2. Lists of enriched GO Biological Processes for differentially expressed transcripts and proteins across all tested comparisons.

Supplemental Table 3. Lists of enriched GO Biological Processes for differentially expressed genes from the RNA-Seq analysis.

Supplemental Table 4. List of top statistically significant upstream regulators obtained by IPA and their targets.

Supplemental Table 5. List of mouse and human qPCR primers.

Mouse	Forward	Reverse
Aspm	5’-TTCTATGACGAACGCTGGAAG-3'	5’-CTTTCCGCTCCCAAAACAAG-3'
Cdkn1a	5’-CAGATCCACAGCGATATCCAG-3'	5’-AGAGACAACGGCACACTTTG-3'
Clpx	5’-ACAAATACTGACCGAGCCAC-3'	5’-TTCTTTCCAGGGCCAATCTC-3'
Gls	5’-GTGGTTTCTGCCCAATTACTG-3'	5’-CCCAGCAACTCCAGATTTTG-3'
Mcm2	5’-AAAGCATCTCCATCTCCAAGG-3'	5’-GGCTCTGTGAGGTCTACATTC-3'
Ppm1b	5’-CTTTCTACCTTCGCCCCAG-3'	5’-ATCTTCCATTTCTACTCTCCATCC-3'
SnrnP27	5’-CTGGAGGGTAAAACAGAGGAAG-3'	5’-AGACACGTTGATGGCATAGG-3'
Tob2	5’-CCTCAGCTACAACCTGAATACC-3'	5’-TCTTTCCCTCTTGCGTTTGG-3'

Human	Forward	Reverse
ASPM	5’-GGACAAAACCCATTATCGCTG-3'	5’-CCCTGTTCCTGCTTTTCCTTC-3'
CDKN1a	5’-TGTCACTGTCTTGTACCCTTG-3'	5’-GGCGTTTGGAGTGGTAGAA-3'
CLPX	5’-GTGCTTTCAGTTGCTGTGTAC-3'	5’-TCATCCTCCCGTCTTCTTATTTC-3'
GLS	5’-TTCCAGAAGGCACAGACATG-3'	5’-GGCTCAGTACTCTTTCACCAG-3'
MCM2	5’-ATTTCGTCCTGGGTCCTTTC-3'	5’-CGCTGGTAGTTCTGATAGATGG-3'
Ppm1b	5’-CCCTTGCCTCAGATTTATTGC-3'	5’-TTCCATTTCCACTCTCCATCC-3'
SMN2 FL	5’-GCTTTGGGAAGTATGTTAATTTCA-3'	5’-CTATGCCAGCATTTCTCCTTAATT-3'
SMN2 Total	5’-GCGATGATTCTGACATTTGG-3'	5’-GGAAGCTGCAGTATTCTTCT-3'
SnRNP27	5’-AGAAACAAAGAGCAAAGAACGG-3'	5’-TTTACAGAGCCATCCACCTTC-3'
Tob2	5’-AGCTACAACCTGAACACCATG-3'	5’-TCTCTTTTCTGTGGTCTTGGG-3'
